# An Expanded Genomic Representation of the Phylum Cyanobacteria

**DOI:** 10.1093/gbe/evu073

**Published:** 2014-05-02

**Authors:** Rochelle M. Soo, Connor T. Skennerton, Yuji Sekiguchi, Michael Imelfort, Samuel J. Paech, Paul G. Dennis, Jason A. Steen, Donovan H. Parks, Gene W. Tyson, Philip Hugenholtz

**Affiliations:** ^1^Australian Centre for Ecogenomics, School of Chemistry and Molecular Biosciences, The University of Queensland, St Lucia, Queensland, Australia; ^2^Advanced Water Management Centre, The University of Queensland, St Lucia, Queensland, Australia; ^3^Biomedical Research Institute, National Institute of Advanced Industrial Science and Technology (AIST), Ibaraki, Japan; ^4^Institute for Molecular Bioscience, The University of Queensland, St Lucia, Queensland, Australia; ^5^Present address: School of Agriculture and Food Sciences, The University of Queensland, St Lucia, Queensland, Australia

**Keywords:** Melainabacteria, culture-independent, evolution, photosynthesis

## Abstract

Molecular surveys of aphotic habitats have indicated the presence of major uncultured lineages phylogenetically classified as members of the Cyanobacteria. One of these lineages has recently been proposed as a nonphotosynthetic sister phylum to the Cyanobacteria, the Melainabacteria, based on recovery of population genomes from human gut and groundwater samples. Here, we expand the phylogenomic representation of the Melainabacteria through sequencing of six diverse population genomes from gut and bioreactor samples supporting the inference that this lineage is nonphotosynthetic, but not the assertion that they are strictly fermentative. We propose that the Melainabacteria is a class within the phylogenetically defined Cyanobacteria based on robust monophyly and shared ancestral traits with photosynthetic representatives. Our findings are consistent with theories that photosynthesis occurred late in the Cyanobacteria and involved extensive lateral gene transfer and extends the recognized functionality of members of this phylum.

## Introduction

Cyanobacteria are recognized primarily for oxygenic photosynthesis (Nelson and Ben-Shem 2004), a feature that is thought to be common to all members of this phylum. However, oxygenic photosynthesis is widely thought to have originated after anoxygenic photosynthesis, likely well after the primary diversification of bacterial phyla (Hohmann-Marriott and Blankenship 2011). This suggests Cyanobacteria as a primary bacterial line of descent predate oxygenic photosynthesis. Consistent with this inference is the relatively shallow phylogenetic depth circumscribed by photosynthetic cyanobacteria compared with other bacterial phyla based on comparative rRNA analyses (table 2 in Dojka et al. 2000).

Cultured Cyanobacteria are categorized into five subsections on phenotypic grounds according to the botanical code (Rippka et al. 1979). However, over the past decade, 16S rRNA-based culture-independent molecular surveys have greatly increased our awareness of the phylogenetic breadth of the Cyanobacteria with the identification of additional major lines of descent classified as YS2/4C0d-2, mle1-12, SM2F09, SM1D11, and ML635J-21 in Greengenes (McDonald et al. 2012) and Silva (Quast et al. 2013). These deep-branching Cyanobacteria have been detected according to Greengenes and Silva classifications (McDonald et al. 2012; Quast et al. 2013) in numerous environments, including drinking water (Williams et al. 2004), grassland soil (Cruz-Martinez et al. 2009), wastewater treatment plants (Liu et al. 2006), and human and animal guts (Ley et al. 2006). Many of these habitats are aphotic, which suggests that a large number of organisms phylogenetically defined as Cyanobacteria are nonphotosynthetic.

Recently, Di Rienzi et al. (2013) obtained five near-complete genomes of members of the YS2/4C0d-2 lineage and confirmed the absence of photosynthetic machinery in these representatives. Additionally, comparative analysis of a 16S rRNA gene sequence (GenBank acc. HM038000) obtained from freeze-dried cells of *Vampirovibrio chlorellavorus* ATCC 29753 (Gromov and Mamkaeva 1980) indicates that this bacterium is a member of the SM1D11 lineage. The original description of this organism provided no indication of photosynthetic capability, further supporting absence of photosynthesis in basal cyanobacterial lineages. Di Rienzi and colleagues proposed a new phylum, Melainabacteria (“Greek nymph of dark waters”), for YS2/4C0d-2 and related basal lineages, given their deeply branching position relative to photosynthetic cyanobacteria and due to their lack of photosynthetic genes (Di Rienzi et al. 2013). To further explore the inferred properties of the Melainabacteria group and to assess whether they should be excluded from the cyanobacterial phylum, we obtained six near-complete genomes representing a broader phylogenetic coverage of the Melainabacteria (representing YS2/4C0d-2, mle1-12, and SM2F09). Comparative analyses of these genomes corroborate the assertion that the Melainabacteria is a nonphotosynthetic lineage; however, they are robustly monophyletic with the photosynthetic cyanobacteria with which they share inferred common ancestral traits, such as cell envelope structure. We therefore suggest that the Melainabacteria represent a class within the Cyanobacteria extending the recognized metabolic capacity of this phylum.

## Materials and Methods

A schematic of the workflow used in this study is presented in supplementary figure S1, Supplementary Material online, with details as follows:

### Sample Collection and DNA Extraction

Fecal samples from a 12-year-old make koala (*Phascolarctos cereus*) named Zagget was collected in sterile 50-ml falcon tubes on 12 May, 2011 (Zag_T1), 28 July, 2011 (Zag_T2), and 24 November, 2011 (Zag_T3), at Lone Pine Koala Sanctuary, Brisbane, Australia. Ethics approval for the collection of koala feces was obtained from the Animal Welfare Unit, The University of Queensland, under ANRFA/074/11 “A Study into Koala Hindgut Microbiology*.*” Samples were snap frozen in dry ice mixed with ethanol at the time of sampling and then transferred to −80 °C until further processing. Genomic DNA was extracted from feces using a MP-BIO FASTSPIN spin kit for soil (MP Biomedicals, Solon, OH) according to manufacturer’s instructions with the exception of two extra ethanol washes (supplementary table S1, Supplementary Material online).

Activated sludge was sampled from two, 4-l enhanced biological phosphorus removal (EBPR) sequencing batch reactors seeded from Thornside Wastewater Treatment Plant, Queensland, on February 16, 2011. The first reactor (EBPR1) was operated on 6-h reaction cycles of 120-min anaerobic phase, 180-min aerobic phase, 20-min settling, 20-min decant (1-l volume removed from the reactor supernatant), and 14-min pre-feed oxygen purge. At the end of each cycle, 1 l of nutrient solution containing 800 mg/l acetate and 40 mg/l phosphate (20:1 COD/P) was added over a period of 6 min (described in detail in Lu et al. [2006]). The second reactor (EBPR2) was operated under identical conditions with the exception that the anaerobic phase lasted 60 min, and the nutrient solution was added over a period of 60 min compared with 6 min for EBPR1. Mixed liquor was collected 90 min into the aerobic phase and microbial biomass was concentrated by centrifugation at 4,000 rpm for 2 min. Samples were collected at six timepoints from EBPR1 (EBPR1_T1 – EBPR1_T6) and at three timepoints from EBPR2 (EBPR2_T1 – EBPR2_T3) (supplementary table S1, Supplementary Material online). DNA was extracted from ∼500 mg (wet weight) of biomass with the MP-BIO FASTSPIN spin kit for soil according to the manufacturer’s instructions (MP Biomedicals).

Methanogenic sludge samples were taken from a full-scale upflow anaerobic sludge blanket (UASB) reactor treating a high-strength organic wastewater discharged from a food-processing factory (Yamada et al. 2011). Two samples of the UASB sludge (A1 and A2) were taken on different dates (A1, December 25, 2012; A2, September 16, 2010). The A1 sample was further subsampled into two parts (flocculant sludge [F1] and granular sludge [G1]) by gravimetric settlement. Four samples in total (A1, A2, G1, F1) were used for sequencing. DNA was extracted by a bead-beating method as described previously (Lu et al. 2006) (supplementary table S1, Supplementary Material online).

### Community Profiling of Koala Feces and EBPR Samples

The V6–V8 regions of the 16S rRNA gene was amplified using fusion primers containing 454 adaptor sequences ligated to the primers 926F (5′-AAACTYAAAKGAATTGRCGG-3′) and 1392R (5′-ACGGGCGGTGTGTRC-3′) (Matsuki et al. 2002). Multiplex identifiers consisting of five nucleotides were incorporated in the 1392R primer to allow for multiplexing. Fifty-microliter PCR reactions were prepared containing 20 ng of template DNA, 5 μl of 10× buffer (Fisher Biotec, Wembley, Australia), 1 μl of 10 mM deoxyribonucleotide triphosphate (dNTP) mix (Fisher Biotec) 1.5 μl bovine serum albumin (BSA) (Fisher Biotech), 4 μl 25 mM MgCl_2_ (Fisher Biotec), 1 μl of each 10 µM primer, and 1 U of Taq polymerase (Fisher Biotec). Cycling conditions were 95 °C for 3 min, followed by 30 cycles of 95 °C for 30 s, 55 °C for 30 s, and 74 °C for 30 s followed by a final extension of 74 °C for 10 min. Following amplification, PCR products for each sample were purified using the Agencourt AMPure XP PCR purification system (Beckman-Coultier) and quantified using the Qubit Fluorometer (Invitrogen, Carlsbad, CA). Amplicons were sequenced from the reverse primers using the Roche 454 GS-FLX Titanium platform at the Australian Centre for Ecogenomics, University of Queensland, Australia (ACE, UQ). Sequence data generated were demultiplexed and processed using a modified version of the QIIME pipeline (Caporaso et al. 2010), which uses Acacia 1.50 (app v 2.0.0) (Bragg et al. 2012) to correct homopolymer errors (modified pipeline is available at https://github.com/Ecogenomics/APP, last accessed April 19, 2014). Sequences were clustered at 97% sequence identity, and the taxonomy of the representatives from each operational taxonomic unit (OTU) was assigned using blastn v. 2.2.26 (Johnson et al. 2008) against the Greengenes database, version 12_10) (DeSantis et al. 2006).

### Community Profiling of UASB Samples

For microbial community structure analysis of the UASB samples, the V4 regions of the 16S rRNA gene were amplified using fusion primers for the Illumina sequencing platform (Caporaso et al. 2012). Multiplex identifiers of 12 nucleotides were incorporated in the M806R primer to allow for multiplexing. PCR conditions were described elsewhere (Lu et al. 2006). Following amplification, PCR products for each sample were purified using the Agencourt AMPure XP PCR purification system (Beckman-Coultier) and quantified using the Qubit Fluorometer (Invitrogen). Amplicons were sequenced from both the forward and reverse primers using the Illumina MiSeq platform and the MiSeq 500 cycles reagent kit v2 (Illumina Inc.). Sequence data generated were demultiplexed and processed using the QIIME pipeline (Caporaso et al. 2010). Sequences were clustered at 97% sequence identity, and the taxonomy of the representatives from each OTU was assigned using blastn (Camacho et al. 2009) against the Greengenes database (DeSantis et al. 2006).

### Paired-End Sequencing

The genomic DNA from Zag_T2, EBPR1_T1, EBPR1_T3, EBPR1_T5, EBPR2_T1, EBPR2_T2, and EBPR2_T3 were sent to Aalborg University, Denmark, where DNA libraries were prepared for sequencing using TruSeq DNA Sample Preparation Kits v2 (Illumina, San Diego, CA) with 2 µg of DNA following the manufacturer’s instructions with nebulizer fragmentation. Library DNA concentration was measured using the QuantIT kit (Molecular Probes, Carlsbad, CA) and paired-end sequenced (2 × 150 bp with an average 250-bp fragment size) on an Illumina HiSeq2000 using the TruSeq PE Cluster Kit v3-cBot-HS (Illumina). The Zag_T2 sample was sequenced on a whole lane of a flowcell and the EBPR1_T1, EBPR1_T3, and EBPR1_T5 were sequenced on a third of a flowcell lane each.

Zag_T1 and Zag_T3, EBPR1_T2, EBPR1_T4, EBPR1_T6 were sequenced at the Institute for Molecular Bioscience, The University of Queensland (IMB, UQ) generating paired-end 150 bp reads (with an average fragment size of 320) using the Nextera DNA Sample Prep kit (Illumina) and the Illumina HiSeq2000 platform. Each Zag sample was sequenced on a quarter of a flowcell lane each, and the EBPR samples were sequenced on a third of a flowcell lane each (supplementary table S1, Supplementary Material online).

DNA extracts from the four UASB sludge samples (A1, A2, F1, F2) were fragmented to 250–400 bp using a Covaris S2 (Covaris, Woburn, MA) and were used for library preparation with a TruSeq sequencing kit (Illumina). Library DNA concentration was measured using the QuantIT kit (Molecular Probes) and paired-end sequenced (2 × 250 bp with an approximate average fragment size of 300 bp) on an Illumina MiSeq system using the MiSeq 500 cycles reagent kit v2 (Illumina Inc.). Each of the four DNA libraries was sequenced in a single MiSeq run (supplementary table S1, Supplementary Material online).

Raw paired-end 2 × 75 bp Illumina data for two male and five female gut metagenome data sets were downloaded from the public MetaHIT database. Details relating to the collection, sequencing, and analysis of the MetaHIT data are provided at http://www.metahit.eu/ (last accessed April 19, 2014) (Qin et al. 2010) (supplementary table S1, Supplementary Material online).

### Sequence Assembly and Population Genome Binning

Paired-end reads for the koala feces, MetaHIT, EBPR, and UASB samples were quality trimmed using CLC workbench v6 (CLC Bio, Taipei, Taiwan) with a quality score threshold of 0.01 (phred score 20) and minimum read lengths as follows; 100 bp for the koala fecal and EBPR samples, 125 bp for the UASB samples, and 50 bp for the MetaHIT samples, in accordance with the read length for each data set. No ambiguous nucleotides were accepted and Illumina sequencing adapters were trimmed if found.

Trimmed sequences for each biome were assembled using CLC’s de novo assembly algorithm, with a kmer size of 63 for koala fecal, EBPR, and UASB samples and a kmer size of 45 for the MetaHIT data.

Population genomes were recovered from the paired-end assemblies using GroopM, version 1.0 with default settings (https://github.com/minillinim/GroopM, last accessed April 19, 2014). Briefly, reads from each sample were mapped onto their corresponding coassemblies (scaffolds ≥ 500 bp; supplementary table S1, Supplementary Material online), and coverage patterns for each scaffold were calculated, transformed and projected onto a three-dimensional plot in which scaffolds from the same population genome would cluster. Integrity of bins was initially confirmed using the GroopM visualization tool.

### Population Genome Completeness and Contamination

All contigs in each population genome bin were translated into six open reading frames (ORFs) and a set of 105 single copy marker genes (a subset of the 111 single copy marker genes widely conserved in Bacteria from Dupont et al. 2012) were identified in the translated data set using HMMER3 (Finn et al. 2011) with default settings and the model-specific Pfam (Punta et al. 2012) and TIGRfam (Haft et al. 2003) thresholds. Completeness was estimated as the percentage of the 105 markers identified in any given population bin, and contamination as the percentage of markers found in >1 copy in a population bin. The marker gene identification and completeness/contamination calculation functions are combined in the software tool CheckM version 0.5.0 (http://ecogenomics.github.io/CheckM/, last accessed April 19, 2014).

**Table 1:** Summary Statistics for Class Melainabacteria Genomes

Population Genome	Number of Scaffolds	Estimated Genome Size (Mb)	%GC	Number of Genes^a^	rRNAs^b^	Estimated Completeness^c^ (%)	Estimated Contamination^c^ (%)	Proposed Candidatus Name	Study
Zag_221	14	1.8	38.5	1,838 (1,799)	16S	100.0	1.0	Gastranaerophilus phascolarctosicola	Present study
Zag_1	322	2.0	34.9	2,194 (2,160)	—	94.3	1.9		Present study
Zag_111	65	2.2	36.7	2,313 (2,257)	5S, 16S 23S	98.1	5.7		Present study
MH_37	157	2.2	34.1	2,402 (2,360)	—	100.0	1.0		Present study
MEL_A1	1	1.9	33.0	1,879 (1,832)	5S, 16S, 23S	100.0	2.9		Di Rienzi et al. (2013)
MEL_B1	21	2.3	35.4	2,269 (2,219)	5S, 16S, 23S	100.0	1.0		Di Rienzi et al. (2013)
MEL_B2	26	2.3	36.3	2,262 (2,215)	5S, 16S, 23S	100.0	1.9		Di Rienzi et al. (2013)
MEL_C1	4	2.1	34.1	2,162 (2,120)	5S, 16S, 23S	100.0	1.9		Di Rienzi et al. (2013)
ACD20^d^	185	2.7	33.5	2,455 (2,325)	5S, 23S	100.0	2.9		Di Rienzi et al. (2013)
EBPR_351	8	5.5	49.4	4,392 (4,342)	5S, 16S, 23S	99.1	7.6	Obscuribacter phosphatis	Present study
UASB_169	67	1.8	27.5	1,917 (1,870)	16S, 23S	94.3	0.0	Caenarcanum bioreactoricola	Present study

^a^Numbers in brackets for number of genes is the number of protein coding genes.

^b^16S rRNA lengths are >1,000 bp.

^c^Estimated completeness and estimated contamination is based on 105 single copy marker genes (a subset of the 111 single copy marker set from Dupont et al. [2012]).

^d^ACD20 is the corrected genome from Albertsen et al. (2013) as the original completeness for ACD20 was 100.0% and original contamination was 107.6%.

### Taxonomic Assignment of Population Genomes

To identify putative representatives of the Melainabacteria among the population bins of a minimum quality threshold (>60% completeness, <10% contamination), we constructed a maximum likelihood tree based on a concatenated set of 83 marker genes with known reference genomes (see Whole-Genome Phylogeny below), including previously reported Melainabacteria population genomes (Di Rienzi et al. 2013).

### Mate Pair Sequencing for Melainabacteria Genome Improvement

Genomic DNA extracted from Zag_T1, Zag_T2, and Zag_T3 were multiple strand-displacement amplified in triplicates using the Illustra Genomiphi V2 DNA amplification kit (GE Healthcare) as per manufacturer’s instructions. One microgram of DNA was used for mate-pair libraries using the Illumina MiSeq sequencing protocol and a gel-free protocol (2–15 kb inserts).

EBPR1_T1, EBPR1_T6, EBPR2_T1, and EBPR2_T3 were sequenced using long-insert mate-pair sequencing according to Illumina’s Nextera protocol. DNA was size selected for 3.5 kb fragments with a standard deviation of 300 bp and further sequenced at IMB, UQ with the Illumina HiSeq platform. Raw mate-pair reads were processed using Prepmate 0.2, removing read pairs, where less than 30 bp remained after trimming the adaptor sequence (https://github.com/ctSkennerton/prepmate, last accessed April 19, 2014). Approximately 50% of the raw reads were retained as valid mate pairs (reads correctly oriented in the reverse-forward direction), resulting in between 16 and 19 million read pairs per sample (supplementary table S1, Supplementary Material online).

For the UASB samples, one of the four samples, A1, was sequenced at the National Institute of Advanced Industrial Science and Technology, Japan (AIST) using the Mate Pair Library Preparation Kit v2 (Illumina) and the MiSeq 500 cycles reagent kit v2 (Illumina) on an Illumina MiSeq system.

Mate-pair sequence data for each sample type (except the public MetaHIT data) were used to scaffold contigs in identified Melainabacteria population genome bins (supplementary table S1, Supplementary Material online) using the default settings of SSPACE v2.0 (Boetzer et al. 2011). Scaffolded assemblies were then checked for completeness and contamination using CheckM.

### 16S rRNA Gene Reconstruction

16S rRNA genes are often difficult to recover via differential coverage binning due to coassembly of rRNA genes present in multiple copies, which distorts their coverage statistics. Therefore, 16S rRNA genes were independently reconstructed from the metagenomic data by extracting read pairs that matched an HMM model of the 16S rRNA gene built using HMMER v3.1b1 from 42,363 bacterial and 1,119 archaeal sequences within the 94% dereplicated Greengenes database released on October 2012 (McDonald et al. 2012). These extracted read pairs were then mapped to the Greengenes database using BWA-MEM v0.7.5a-r405 (Li and Durbin 2010). A read was considered reliably mapped if at least 85% of the read aligned to the reference sequence, and the edit distance of the alignment was at most 10% of the length of the read (e.g., less than 10 for 100 bp reads). Pairs were further filtered to remove any pair where both reads did not correctly map to reference sequences within a branch length of 0.03 as measured over the Greengenes phylogeny.

The remaining pairs were clustered in a greedy manner in order to identify pairs mapping to similar Greengenes reference sequences. Reference sequences were put in ascending order according to the number of pairs mapped to them. Starting with the reference sequence with the highest number of assigned pairs, any pairs assigned to a reference sequence within a branch length of 0.03 to this reference sequence were clustered together and removed from further consideration. This process was repeated until all pairs were assigned to a cluster. Each cluster of 16S pairs was then independently assembled using CLC Workbench v6.5. Using this technique, an additional 16S rRNA gene was recovered from Zag_221 (1,403 bp).

### 16S rRNA Phylogeny

16S rRNA genes from Melainabacteria population genomes were aligned to the standard Greengenes alignment with PyNAST (McDonald et al. 2012). Aligned sequences and a Greengenes reference alignment, version gg_13_5 (ftp://greengenes.microbio.me/ [last accessed April 19, 2014] greengenes_release; [McDonald et al. 2012]) were imported into the phylogenetic software package ARB (Ludwig et al. 2004), and the Melainabacteria sequence alignments were manually corrected using the ARB EDIT tool. For constructing the alignment data of different taxon configurations, representative taxa (>1,300 nt) were selected and their alignment data were exported from ARB with the lane mask filtering, resulting in totals of 402 and 67 taxa for two data sets. Neighbor-joining trees were calculated from the masked alignments with LogDet distance estimation using PAUP*4.0 (Swofford 2003). A further analysis was run with 100 bootstrap replicates. Maximum parsimony trees were calculated using PAUP*4.0 (Swofford 2003). A heuristic search was used with a random stepwise addition sequence of ten replicates and nearest-neighbor-interchange swapping. A further analysis was run with 100 bootstrap replicates. Maximum likelihood trees were calculated from the masked alignments using the Generalized Time-Reversible model with Gamma and I options in RAxML version 7.7.8 (Darling et al. 2014) (raxmlHPC-PTHREADS -f a -k -x 12345 -p 12345 -N 100 -T 4 -m GTRGAMMAI). Bootstrap resampling data (100 replicates) were generated with SEQBOOT in the phylip package (Felsenstein 1989) and were used for 100 bootstrap resamplings. Generated trees were reimported into ARB for visualization.

**Fig. 1.—:**
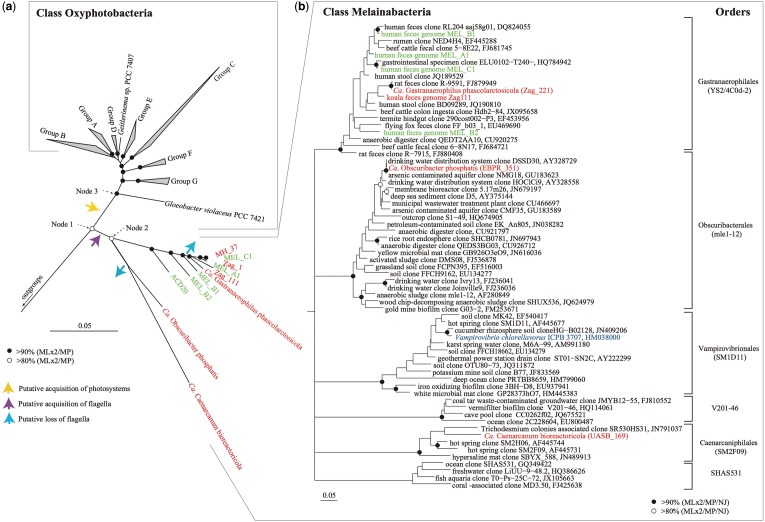
Phylogenetic trees of the phylum Cyanobacteria. (*a*) A maximum likelihood tree of the phylum Cyanobacteria based on the concatenated alignment of 83 phylogenetically conserved proteins (supplementary table S2, Supplementary Material online). Chloroflexi genomes were used as the outgroup (supplementary table S3, Supplementary Material online). Groups A–G for the Oxyphotobacteria were based on the names given by Shih et al*.* (2013). *Candidatus* has been abbreviated to *Ca.* Bootstrap resampling analyses (100 times) with maximum likelihood (ML; using RAxML and FastTree), and maximum parsimony (MP; using PAUP*) methods were performed for different taxon configurations with 38 or 83 conserved proteins (supplementary figs. S1–3, Supplementary Material online), and the phylogenetic robustness (monophyly score) of taxa is indicated at the node: Black circles in the tree represent nodes with >90% bootstrap supports by all calculations, white circles represent nodes with >80% bootstrap supports by all calculations. Putative acquisitions of photosystem and flagella genes are indicated by colored arrows. Genomes in green are representatives from Di Rienzi et al. (2013), and genomes in red are Melainabacteria from this study. (*b*) A maximum likelihood tree based on 16S rRNA genes from the class Melainabacteria obtained by Di Rienzi et al*.* (2013) and this study, together with public representatives from the Greengenes database version 13_05 and Silva 115 database. 16S sequences in green are Melainabacteria representatives from Di Rienzi et al. (2013), the Melainabacteria in red are representatives from this study and the 16S from the cultured representative, *V. chlorellavorus,* is highlighted in blue. Node support values are as described for panel (a). Proposed order level designations within the class Melainabacteria are shown to the right of the figure. A third potential class-level lineage in the Cyanobacteria, ML635J-21 (supplementary fig. S5, Supplementary Material online) is not shown.

### Whole-Genome Phylogeny

Two sets of ubiquitous single copy marker genes were obtained and aligned from eleven draft Melainabacteria population genomes (table 1) and up to 434 complete bacterial and archaeal reference genomes (Markowitz et al. 2012) using HMMER3 (Finn et al. 2011). The first set of 38 markers (Darling et al. 2014) is found in nearly all Bacteria and Archaea, and the second set of 83 markers (supplementary table S2, Supplementary Material online) was derived from a subset of the 111 bacterial marker set (Dupont et al. 2012) based on congruent taxonomic signal as follows. Individual gene trees were constructed using FastTree v2.1.7 (Price et al. 2010) and compared with the Integrated Microbial Genomes (IMG) taxonomy (Markowitz et al. 2012). We used the following measure to quantify the agreement of each node in an unrooted gene tree with a specific clade *c* (e.g., Bacteria, Firmicutes) within the IMG taxonomy:

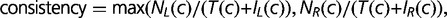

where *T*(*c*) is the total number of genomes from clade *c*, the subscripts *R* and *L* indicate the subset of genomes to the “right” and “left” of the node under consideration, *N_x_*(*c*) is the number of genomes in subset *x* from clade *c*, and *I_x_*(*c*) is the number of genomes in subset *x* not from clade *c*. The consistency of a clade *c* was assigned the highest consistency found over all nodes. Average consistencies were then determined over all clades with at least five genomes independently at the domain, phylum, and class ranks. Gene trees where the average consistency over these three ranks was less than 0.86 were discarded as a sharp dropoff in consistency was observed beyond this threshold.

Ambiguous and uninformative alignment positions were removed from aligned sets of concatenated marker genes using gblocks (Castresana 2000) under default settings, with the exception that a conserved position was not allowed to have gaps in more than half of the sequences. Phylogenetic trees were reconstructed from the two filtered marker gene alignments with outgroup configurations as detailed in supplementary tables S3 and S4, Supplementary Material online. All tree topologies were tested for robustness using the maximum likelihood methods from FastTree version 2.1.7 (JTT model, CAT approximation) (Price et al. 2010), RAxML version 7.7.8 with a JTT and Gamma models (Stamatakis 2006) (raxmlHPC-PTHREADS -f a -k -x 12345 -p 12345 -N 100 -T 8 -m PROTGAMMAJTT), and maximum parsimony method using PAUP*4.0 with heuristic search—a random stepwise addition sequence of ten replicates—and nearest-neighbor-interchange swapping. Generated trees were imported into ARB, where they were rooted, beautified, and grouped for display purposes.

### Melainabacteria Genome Annotation and Metabolic Reconstruction

**Fig. 2.—:**
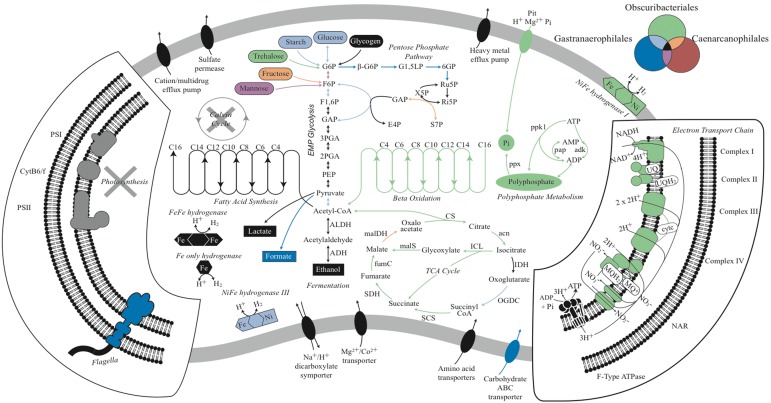
Metabolic reconstruction of Melainabacteria representatives. Metabolic predictions for Gastranaerophilales, Obscuribacteriales, and Caenarcaniphilales representatives based on genome annotations. Names of pathways are italicized, and fermentation products are shown in rectangles. Features identified in one or more of the orders are highlighted by color according to the color key in the top right corner. Missing pathways are shown in gray. The Melainabacteria are missing photosystems I and II, and the Calvin cycle, which are found in Oxyphotobacteria. All Melainabacteria use the EMP pathway to produce fermentation products, and the Obscuribacteriales representative is capable of using both aerobic and anaerobic respiration to produce energy.

The draft Melainabacteria genomes were submitted to IMG/ER (Expert Review) (Markowitz et al. 2009) for automated annotation and manual analysis. KEGG (Kyoto Encyclopedia of Genes and Genomes) maps and gene annotations were used to reconstruct the metabolism of the Melainabacteria representatives, and a composite metabolic cartoon was prepared in Adobe Illustrator CS6 (fig. 2 and supplementary table S5, Supplementary Material online).

Average nucleotide identity was calculated using the ANI calculator with default settings (http://enve-omics.ce.gatech.edu/ani/, last accessed April 19, 2014).

### Protein Family Analysis

**Fig. 3.—:**
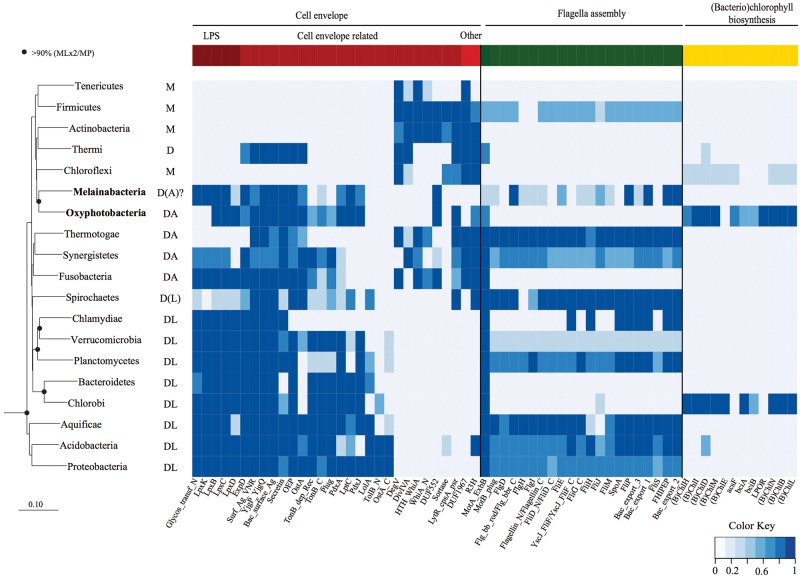
Distribution of key traits across the Cyanobacteria and other bacteria phyla. A maximum likelihood genome tree of the bacterial domain constructed using a concatenated alignment of 38 conserved proteins is shown at the left of the figure for phylogenetic ordering of traits shown in the heat map to the right. Black circles on interior nodes represent affiliations with >90% bootstrap support. Columns in the heat map represent individual gene families (Pfams or GIs; supplementary table S6, Supplementary Material online) grouped into three subsystems of interest; cell envelope, flagella, and (bacterio)chlorophyll biosynthesis. Increasing representation of each gene family in a given phylum or class (percentage of genomes) is shown by increasing depth of color. Cell envelope classification is indicated by the abbreviations to the right of the phylum names: Monoderm (M), Diderm (D), Diderm-LPS (DL), Diderm-Atypical (DA) from Albertsen et al*.* (2013). Note that it is not possible to determine whether the Melainabacteria are diderms or atypical diderms based on sequence data only.

The presence of Pfams and TIGRfams for maximally differentiating cell wall types, as previously described in Albertsen et al. (2013) and flagella assembly (Pallen and Matzke 2006) were identified in the draft Melainabacteria genomes and 2,363 representative phyla using complete bacterial genomes obtained from IMG (v4.0) (Markowitz et al. 2012) (supplementary table S6-S7, Supplementary Material online). Photosynthesis and (bacterio)chlorophyll biosynthesis genes as described in Sousa et al. (2012) (supplementary table S7, Supplementary Material online) were also identified as present or absent by using the BLASTP module (Altschul et al. 1997) in IMG with an e-value of >1e-10 and amino acid identities of ≥25%. Paralogs from the cobalamin pathway or later steps in the bacteriochlorophyll c pathway were removed in accordance with the analysis of Sousa et al. (2012). The color key represents the number of species within a certain phylum that have the Pfams, TIGRfams, or genes versus the total number of complete bacterial genomes obtained from IMG or the draft Melainabacteria genomes. A heat map was constructed in RStudio v0.95.265 (Racine 2012) using gplots (Warnes et al. 2013) and RColorBrewer (Neuwirth 2011) (fig. 3).

COG (Clusters of Orthologous Groups) profiles for each genome were constructed through homology search between ORFs predicted with Prodigal v2.60 (Hyatt et al. 2010) and the 2003 COG database (Tatusov et al. 2003). Homology was assessed with BLASTP v2.2.26+ (Camacho et al. 2009) using an e-value threshold of 1e-5 and a percent identity threshold of 30%. The relative percentage of each COG was calculated in relation to the total number of ORFs predicted for each genome. All statistical plots and analyses were conducted using STAMP v2.0.1 (Parks and Beiko 2010).

## Results and Discussion

During ongoing culture-independent molecular surveys, 16S rRNA phylotypes belonging to basal cyanobacterial lineages were identified in a number of habitats. These included fecal samples collected from a geriatric male koala (*Phascolarctos cinereus*; Zagget), a lab-scale sequencing batch reactor performing EBPR and a UASB reactor treating a high-strength organic wastewater discharged from a food processing factory (see sampling details below). In parallel, public metagenomic data sets of human fecal samples containing members of YS2/4C0d-2 (Qin et al. 2010) were reanalyzed with the goal of obtaining additional genomes from this lineage.

Three distantly related (88% 16S rRNA gene identity) phylotypes belonging to the YS2/4C0d-2 lineage were detected in the koala feces, a representative of mle1-12 was identified in the EBPR bioreactor and a representative of SM2F09 was identified in the UASB reactor (fig. 1). Although Melainabacteria are typically found in low abundance, these phylotypes comprised up to 6.7%, 4.2%, and 1.7% of the koala fecal, EBPR, and UASB microbial communities, respectively. Samples with the highest relative abundance of Melainabacteria were chosen for deep metagenomic sequencing to improve the likelihood of obtaining near-complete population genomes for comparative analysis. The relative abundance of the Melainabacteria in the MetaHIT shotgun data sets was estimated to be up to 2.6% by direct classification of 16S rRNA genes in the shotgun data sets.

### Recovery of Melainabacteria Population Genomes

Koala fecal samples were collected from three timepoints from the same koala over a period of 6 months and sequenced to produce 90.7 Gb of metagenomic data. Two EBPR reactors were sampled six times and three times, respectively, over a period of 7 months, producing 211.6 Gb, and the UASB reactor was sampled twice producing 31.5 Gb of metagenomic data (supplementary table S1, Supplementary Material online). Human fecal metagenomes from healthy Danish individuals (two male and five female) were obtained from the public repository (http://www.ebi.ac.uk/ena/home [last accessed April 19, 2014], study accession number ERP000108) comprising a total of 21.6 Gb. Multiple data sets from the same sample types were coassembled, which produced between 3,139 and 148,338 contigs with an N50 of 1.4 kb–4.6 kb. Population genomes were extracted from the assemblies using differential coverage binning of each set of related metagenomes. This approach leverages differences in relative abundance of target populations between two or more related samples to identify contigs with covarying coverage (Albertsen et al. 2013; Sharon et al. 2013). Population genomes obtained using this method were taxonomically assigned by placement in concatenated gene trees comprising all finished IMG reference genomes (Markowitz et al. 2012) (supplementary table S2, Supplementary Material online; see below). Six population genomes were found to form a monophyletic lineage together with the reference cyanobacterial genomes (fig. 1*A*). These comprised one mle1-12 representative from the EBPR (EBPR_351), one SM2F09 representative from the UASB reactor (UASB_169), and four YS2/4C0d-2 representatives from koala and human feces (Zag_1, Zag_111, Zag_221, and MH_37) consistent with 16S rRNA gene amplicon community profiling. Analysis of 16S rRNA genes recovered from four of the six population genomes also confirmed that they are members of the Melainabacteria (table 1 and fig. 1*B*). Further sequencing using long-insert (2–15 kb) libraries of the EBPR, UASB reactor, and koala feces were used to improve the quality of the draft population genomes (supplementary table S1, Supplementary Material online). The completeness and degree of contamination of the improved genomes was estimated by determining the presence and number of copies of 105 conserved single-copy bacterial marker genes (Dupont et al. 2012). All population genomes had >90% estimated completeness (>97 of the 105 conserved single-copy bacterial marker genes) and <10% contamination (multiple copies of genes expected to be present as single copies) (table 1).

### An Expanded Phylogenetic Classification of the Phylum Cyanobacteria

We began analysis of the Melainabacteria genomes by constructing phylogenetic trees based on two concatenated alignments of broadly conserved single copy marker genes (see Materials and Methods; supplementary table S2, Supplementary Material online, and Darling et al. [2014]). The ingroup comprised 81 reference cyanobacterial genomes and 11 Melainabacteria genomes; six determined in the present study and the five most complete genomes obtained in the Di Rienzi et al*.* (2013) study (fig. 1*A* and supplementary table S3, Supplementary Material online). We evaluated the monophyly of the photosynthetic Cyanobacteria and Melainabacteria groups using up to 377 outgroup genomes representing 28 phyla (supplementary figs. S2–S4 and table S4, Supplementary Material online). In all cases, the evolutionary association between the two groups was reproducibly resolved with >80% bootstrap support (node 1, fig. 1*A*). Di Rienzi et al*.* (2013) concluded that the two groups are sister phyla rather than a single phylum based on a combined divergence slightly greater than a recommended threshold of 85% 16S rRNA gene sequence identity for distinguishing new phyla (Hugenholtz et al. 1998). However, the primary criterion for defining a new phylum is not satisfied in this instance, that is, that the lineage is reproducibly unaffiliated with existing phyla (Hugenholtz et al. 1998) according to both 16S rRNA and genome-level phylogenies. There are well-known examples of reproducibly associated (sister) phyla in the bacterial domain grouped into superphyla, such as the PVC (Planctomycetes, Verrucomicrobia, Chlamydiae) superphylum (Wagner and Horn 2006), which arguably should be consolidated into single phyla according to the Hugenholtz et al. (1998) definition. We suggest that the PVC and other superphyla are historical artifacts that should be consolidated into phyla to provide a more naturalistic taxonomy based on reproducible divergence points in evolutionary trees.

Several inferred features beyond the evolutionarily conserved core set of genes used to construct the genome tree are consistent with a common ancestry between the photosynthetic cyanobacteria and Melainabacteria (see below). Therefore, we propose that the phylum Melainabacteria should be reclassified as the class Melainabacteria within the phylum Cyanobacteria. Me.lai.na.bac.te.ria. Gr. n. Melaina, a nymph in Greek mythology, who presides over dark subterranean aspects; N.L. masc. n. *bacter* (from Gr. n. *baktron*), a rod; suff *-ia* ending to denote a class; N.L. fem. pl. n. *Melainabacteria* class of bacteria found in the dark.

The node defining the Melainabacteria in the concatenated gene alignment tree (node 2, fig. 1*A*) was supported in all analyses with >80% confidence consistent with the genome-based analysis of Di Rienzi et al*.* (2013). The population genomes formed three primary lines of descent within the Melainabacteria, with the human and koala gut genomes and groundwater genome ACD20 (Di Rienzi et al. 2013) forming a monophyletic cluster. The 16S rRNA-based inference provides only modest support for the monophyly of the Melainabacteria (fig. 1B and supplementary fig. S5, Supplementary Material online), and indeed several of the robustly monophyletic groups therein are classified as primary cyanobacterial lines of descent (classes) in Greengenes and Silva (McDonald et al. 2012; Quast et al. 2013). The higher resolution afforded by the genome sequences suggests that these lineages should be classified as orders within the class Melainabacteria (fig. 1). We propose names for four of these orders based on habitat and analysis of the population genomes (see below), and the recognition of *V. chlorellavorus* in the SM1D11 lineage (fig. 1). The location of the ACD20 genome could not be determined within the 16S rRNA gene tree (fig. 1*B*) as it lacks a 16S rRNA gene sequence, but genome trees based on a refined binning of this population (Albertsen et al. 2013) (table 1) indicate that it is basal and monophyletic with the order, Gastranaerophilales (YS2/4C0d2; fig. 1*A* and supplementary figs. S2 and S3, Supplementary Material online). An additional distantly related cyanobacterial lineage ML635J-21 (named in the Greengenes and Silva databases; McDonald et al. 2012; Quast et al. 2013) is currently not represented by a sequenced genome and may represent another class-level lineage within the Cyanobacteria (supplementary fig. S5, Supplementary Material online), highlighting the need for further genomic exploration of the cyanobacterial phylum.

Our analyses show that the photosynthetic cyanobacteria are robustly monophyletic within the expanded context of the phylum Cyanobacteria (node 3, fig. 1*A*). We therefore propose to reinstigate the name Oxyphotobacteria (Gibbons and Murray 1978) to describe all photosynthetic cyanobacteria (including chloroplasts) in a single class. Gr. adj. *oxus*, acid or sour and in combined words indicating oxygen; Gr. n. *phos photos*, light; Gr. n. *baktêria*, staff, cane; suff. -*ia*, ending to denote a class; N.L. neut. pl. n. *Oxyphotobacteria*, light-requiring bacteria that produce oxygen. The name implies that the class is able to photosynthesize. We denote the order-level groupings within this class as A to G (fig. 1*A*) in accordance with a recent genome-based analysis (Shih et al. 2013). Oxyphotobacteria are still classified primarily on morphological grounds into five subsections (Rippka et al. 1979) despite clear incongruencies between phylogenetic reconstructions and morphological complexity (Shih et al. 2013). Therefore, it is likely that the order- and family-level groupings within the Oxyphotobacteria will be reclassified on phylogenetic grounds with a concomitant widespread reclassification of cyanobacterial strains once this group is no longer under the jurisdiction of the Botanical Code (Komárek 2010).

### Inferred Metabolism of Melainabacteria Genomes

Di Rienzi et al. inferred metabolic properties of the class Melainabacteria based on comparative analyses of draft population genomes belonging to only one order, the Gastranaerophilales (fig. 1). We substantially increase the phylogenetic coverage of the Melainabacteria in the present study by recovery of population genomes spanning three of the six identified orders (fig. 1*B*). Expanded genomic representation should provide a more balanced overview of the metabolic properties of this class including features in common with, or distinct from, the Oxyphotobacteria. We begin by proposing Candidatus species for the most complete genomes in each of the three orders obtained in this study and describe their inferred metabolic properties below.

The most complete Gastranaerophilales genome with the least number of scaffolds, Zag_221, was selected as the Candidatus species representative of the group, *Candidatus* Gastranaerophilus phascolarctosicola (table 1 and fig. 1). *Gastranaerophilus phascolarctosicola* (Gas.tra.nae.ro.phi’lus. Gr. n. *gaster* stomach; Gr. pref. *an*-, not; Gr. masc. n. *aer*, air; L. masc. adj. *philus* [from Gr. adj.*philos*], friend, loving; *Gastranaerophilus* a bacterium-loving anaerobic gastric environments. “phas.co.larc.to.si.co.la.” N.L. *Phascolarctos* the name of koala; L. suffix *-cola* inhabitant, dweller; N.L. masc. n. *phascolarctosicola* hiding in the belly of a koala).

The genome size and GC content range of the four Gastranaerophilales genomes were in accord with the Di Rienzi *et al.* population genomes from this order (table 1). Members of this group have small streamlined genomes ranging in size from 1.8 to 2.3 Mb, with the exception of ACD20, which is 2.7 Mb after binning refinement (Albertsen et al. 2013). The Gastranaerophilales genomes recovered from the koala and human feces in the present study support the assertion (Di Rienzi et al. 2013) that this lineage comprises obligate fermenters missing the genes necessary for aerobic and anaerobic respiration as well as the tricarboxylic acid (TCA) cycle (fig. 2 and supplementary table S5, Supplementary Material online). Instead, all Gastranaerophilales genomes contain the Embden–Meyerhof–Parnas (EMP) pathway, capable of converting glucose, mannose, starch, or glycogen into lactate, ethanol, and/or formate (fig. 2). All representative genomes have the potential to produce riboflavin, nicotinamide, biotin, dehydrofolate, and pantoate as found previously (Di Rienzi et al. 2013).

Di Rienzi et al*.* highlighted the presence of FeFe hydrogenases in their human gut population genomes, speculating that these organisms are hydrogen-producing anaerobes in syntrophic interactions with hydrogenotrophic methanogens or acetogens. We also identified FeFe hydrogenases in the MH_37 genome obtained from the human gut, but in contrast found Fe-only or NiFe hydrogenases in koala gut Gastranaerophilales genomes (supplementary fig. S6, Supplementary Material online). It is possible that the less oxygen-sensitive NiFe hydrogenase would allow members of this order to colonize the jejunum as well as the more anaerobic colon (Quigley and Quera 2006).

Di Rienzi et al. also reported that the Melainabacteria are flagellated based on the presence of a complete set of flagella genes in three of their draft Gastranaerophilales genomes (MEL_B1, MEL_B2, and ACD20). Of the nine Gastranaerophilales genomes available (table 1), only these three had complete flagella gene sets, the remainder having only a subset of genes that would not encode a functional flagellum (supplementary fig. S7, Supplementary Material online). We infer that flagella were present in the ancestor of the class Melainabacteria and subsequently lost on at least two occasions based on monophyly of flagella genes common to the Gastranaerophilales and Caenarcaniphilales (fig. 1*B* and supplementary fig. S7, Supplementary Material online). Moreover, there appears to have been a subsequent loss of functional flagella in *G. phascolarctocola* and relatives (supplementary fig. S7, Supplementary Material online), indicating the presence of nonmotile members of this order in animal gut habitats.

Based on a suggested species threshold of 95% average nucleotide identity (Goris et al. 2007), three of the Gastranaerophilus genomes can be considered to belong to the same species (table 1 and supplementary fig. S8, Supplementary Material online). Two of these genomes were recovered from humans (MH37 and MEL_C1) and the third from a koala (Zag_1) despite the structural and physiological differences between the human and koala gut. The majority of genes that differ between these genomes are hypothetical proteins or phage associated (supplementary fig. S8, Supplementary Material online), typical of differences seen between strains belonging to the same species.

*Candidatus* Obscuribacter phosphatis (Ob.scur.i.bac.ter. L. adj. *obscurus* dark; N.L. masc. n. *bacter* (from Gr. n. *baktron*), a rod; *Obscuribacter* a bacterium found in the dark. “phos.pha.tis.” N.L. n. *phosphatis*, phosphate; N.L. *phosphatis* accumulating phosphate) EBPR_351 representing the order Obscuribacterales (fig. 1 and table 1) is conspicuous among the Melainabacteria genomes because of its larger size (5 Mb) and associated metabolic versatility. *Obscuribacter phosphatis* contains the genes necessary for polyphosphate metabolism, including a low-affinity inorganic phosphate transporter (PiT), polyphosphate kinase 1 (used to synthesize or degrade polyP while consuming or generating, respectively, ATP directly), polyphosphate kinase 2 (degrades polyP producing GTP from GDP), exopolyphosphatase (degrades polyP in a nonreversible reaction that does not generate ATP directly), polyphosphate:AMP phosphotransferase and adenylate kinase (Seviour and Nielsen 2010). *Obscuribacter phosphatis* has the capacity for aerobic and anaerobic respiration, and fermentation, allowing it to function during both the oxic and anoxic phases of EBPR (Blackall et al. 2002). It contains genes encoding a complete respiratory chain including Complexes I, II, III, and IV and an F-Type ATPase (fig. 2). Di Rienzi et al*.* concluded that the Melainabacteria lack electron transport chains and are therefore incapable of respiration. This highlights the dangers of inferring phylum- or class-level functionality based on limited phylogenetic sampling of the lineage.

Like the Gastranaerophilales, *O. phosphatis* has the capability to metabolize a wide range of simple carbohydrates via the EMP pathway and also fatty acids via the beta-oxidation pathway (fig. 2). Under oxic conditions, we predict that *O. phosphatis* will fully oxidize one or more of these substrates via the TCA cycle, feeding nicotinamide adenine dinucleotide (NADH) into the electron transport chain with a cbb3-type cytochrome as the terminal oxidase. This family of cytochromes is typically used in microaerophilic conditions (Kulajta et al. 2006), suggesting that *O. phosphatis* may be found within flocs where oxygen concentrations are lower (Albertsen et al. 2013). Under anoxic conditions, we predict that it performs either respiration with nitrate as the terminal electron acceptor or, in the absence of nitrate, mixed-acid fermentation with the potential to produce ethanol, lactate, formate, succinate, CO_2_, and H_2_ (fig. 2). The presence of these metabolic pathways suggests that *O. phosphatis* has adapted to more dynamic environments (requiring greater metabolic plasticity) with “feast-famine” nutrient cycles such as those artificially imposed in EBPR bioreactors.

*Candidatus* Caenarcanum bioreactoricola (“Caen.arc.an.um” L. neut. n. *caenum* mud, sludge; L. neut. n. *arcanum* secret, hidden; N.L. neut. n. *Caenarcanum* a bacterium hidden in sludge. “bio.re.ac.to.ri.co.la.” L. suffix *-cola* inhabitant, dweller; N.L. masc. n. *bioreactericola* living in a bioreactor) UASB_169 representing the order Caenarcaniphilales, has an estimated genome size of ∼2 Mb and a remarkably low GC content of 27.7%, the lowest GC content yet reported for Cyanobacteria. Similar to the Gastranaerophilales genomes, *C. bioreactoricola* lacks the genes necessary for aerobic and anaerobic respiration, as well as the TCA cycle, suggesting that it has a streamlined metabolism only producing energy via fermentation with ethanol and lactate as the main fermentation products. *C. bioreactoricola* contains the subunits for Fe-only hydrogenase and the potential to produce hydrogen as a by-product from the fermentation process. Like the Gastranaerophilales, *C. bioreactoricola* may also be a hydrogen producer living in syntrophy with methanogens or acetogens in the bioreactor, as microcolonies of syntrophic bacteria are often observed in the granules from UASB systems and electron transfer in these microcolonies is thought to mostly occur through interspecies hydrogen transfer (Schmidt and Ahring 1996).

### Emergence of Photosynthesis in the Cyanobacteria

Melainabacteria resemble Oxyphotobacteria in their cell envelope gene complement comprising genes indicative of a Gram-negative (diderm) cell wall (fig. 3). This includes genes for the biosynthesis of Lipid A for the production of lipopolysaccharide (LPS), as previously reported for members of the Gastranaerophilales (Di Rienzi et al. 2013). Oxyphotobacteria also have unusual cell envelope components for Gram-negative bacteria including porins (*somA, somB*), which are thought to help anchor the outer membrane to the peptidoglycan layer (Hansel et al. 1998; Hoiczyk and Hansel 2000). All Melainabacteria have closely related homologs to the oxyphotobacterial *somA* and *somB* genes suggesting that their cell envelopes comprise similar porins.

Di Rienzi et al. highlighted the presence of putative circadian rhythm (*rpaA* and *rpaB*) and light-response (*nblS*) regulators in the Gastranaerophilales, which are diagnostic of Cyanobacteria. We identified orthologs of these genes in all three orders of the Melainabacteria, suggesting that these are uniquely ancestral features of the phylum. Together with the unambiguous phylogenetic placement of the Melainabacteria within the cyanobacterial radiation based on comparative analysis of highly conserved marker genes (fig. 1), the conservation of features characteristic of Oxyphotobacteria in the Melainabacteria further support a common ancestry and the proposal for a single phylum.

The most conspicuous difference between the Melainabacteria and Oxyphotobacteria is the absence of chlorophyll biosynthesis genes in the former (fig. 3), consistent with previous findings (Di Rienzi et al. 2013). All subunits of photosystems I and II are absent from the Melainabacteria genomes sequenced in this study. Another trait characteristic of photosynthetic cyanobacteria, carbon fixation, is similarly absent in the Melainabacteria (fig. 2), indicating that these organisms do not engage in a photoautotrophic lifestyle. Instead, members of this lineage appear to be chemoheterotrophs with diverse functionality.

The idea of nonphotosynthetic Cyanobacteria is contrary to the prevailing dogma that all members of this phylum are photosynthetic (Shih et al. 2013). However, this should not be a controversial conclusion given that photosynthesis is only found in sublineages of several other phyla such as the Proteobacteria, Firmicutes, Acidobacteria, and Chloroflexi (Bryant and Frigaard 2006). The complete absence of photosynthetic apparatus in the Melainabacteria suggests that the Oxyphotobacteria acquired photosystems after diverging from the common ancestor of the Melainabacteria (fig. 1). This is consistent with the inferences that photosynthesis genes have an extensive history of lateral transfer (Hohmann-Marriott and Blankenship 2011) and that photosynthesis developed late in the Cyanobacteria (Xiong et al. 2000).

The acquisition of oxygenic photosynthesis in the Oxyphotobacteria had a profound impact not only on the biosphere (Hohmann-Marriott and Blankenship 2011) but also left imprints in their genomes that are now apparent by contrasting with their newly sequenced nonphotosynthetic relatives. For example, there was a great expansion of ATP-driven transport systems in the Oxyphotobacteria likely for acquiring bicarbonate (COG0600/0715/1116) and iron (COG0609/1629/0735) necessary for photosynthesis and respiration (supplementary fig. S9, Supplementary Material online). The additional energy available to Oxyphotobacteria via oxygenic photosynthesis may also explain the widespread acquisition of energy-intensive biosynthetic pathways such as secondary metabolite synthesis (Shih et al. 2013) (supplementary fig. S10, Supplementary Material online).

## Conclusion

Our findings expand the recognized phylogenomic boundaries of the phylum Cyanobacteria. We infer that the cyanobacterial ancestor was a nonphotosynthetic chemoheterotroph and that photosystems were acquired after divergence of the classes Melainabacteria and Oxyphotobacteria. We suggest that the acquisition of oxygenic photosynthesis resulted in an increase in genome complexity within the Oxyphotobacteria (followed by a subsequent reduction and streamlining in the *Prochlorococcus* lineage; [Partensky and Garczarek 2010]), while the Melainabacteria mostly retained a simpler ancestral metabolism. Consistent with the phylogenetic depth of the Melainabacteria, members of this class occupy a wide range of environmental niches with varied metabolic properties, mostly centered around fermentative lifestyles, that extend the known metabolic diversity of the Cyanobacteria. These include respiratory nitrate reduction (*O. phosphatis*) and flagella-based motility (in some Gastranaerophilales [Di Rienzi et al. 2013]). If the inclusion of *V. chlorellavorus* (Coder and Goff 1986) in the Melainabacteria is confirmed by genomic sequencing, then parasitism can also be added to the known phenotypes of Cyanobacteria. The availability of 11 high-quality draft genomes representing multiple orders within the Melainabacteria (table 1) provides a sound basis for further investigations into this fascinating group, for example, via targeted visualization (Moter and Göbel 2000) and genome-directed isolation (Tyson et al. 2005; Pope et al. 2011).

## Supplementary Material

Supplementary figures S1–S10 and tables S1–S7 are available at *Genome Biology and Evolution* online (http://www.gbe.oxfordjournals.org/). 

Supplementary Data
