# Supplementary material for: An Expanded Genomic Representation of the Phylum Cyanobacteria
Source: Genome Biol Evol. 2014 May 2;6(5):1031–45. doi: 10.1093/gbe/evu073 (PMC4040986; doi:10.1093/gbe/evu073)
Supplement: Supplementary Data [file supp_6_5_1031__index.html]

An Expanded Genomic Representation of the Phylum Cyanobacteria — An Expanded Genomic Representation of the Phylum Cyanobacteria — Supplementary Data 

# An Expanded Genomic Representation of the Phylum Cyanobacteria

## Supplementary Data

files

**Files in this Data Supplement:**

- Supplementary Data - pdf file
- Supplementary Data - pdf file
