# Supplementary material for: An Expanded Genomic Representation of the Phylum Cyanobacteria
Source: Genome Biol Evol. 2014 May 2;6(5):1031–45. doi: 10.1093/gbe/evu073 (PMC4040986; doi:10.1093/gbe/evu073)
Supplement: Supplementary Data [file supp_evu073_Soo_GBE_Supplementary_Figures.pdf]

Supplementary Figures for

An Expanded Genomic Representation of the Phylum Cyanobacteria

Rochelle M. Soo, Connor T. Skennerton, Yuji Sekiguchi, Michael Imelfort, Samuel J. Paech, Paul G. Dennis, Jason A. Steen, Donovan H. Parks, Gene W. Tyson, and Philip Hugenholtz[†]

[†] Correspondence to: Philip Hugenholtz, p.hugenholtz@uq.edu.au

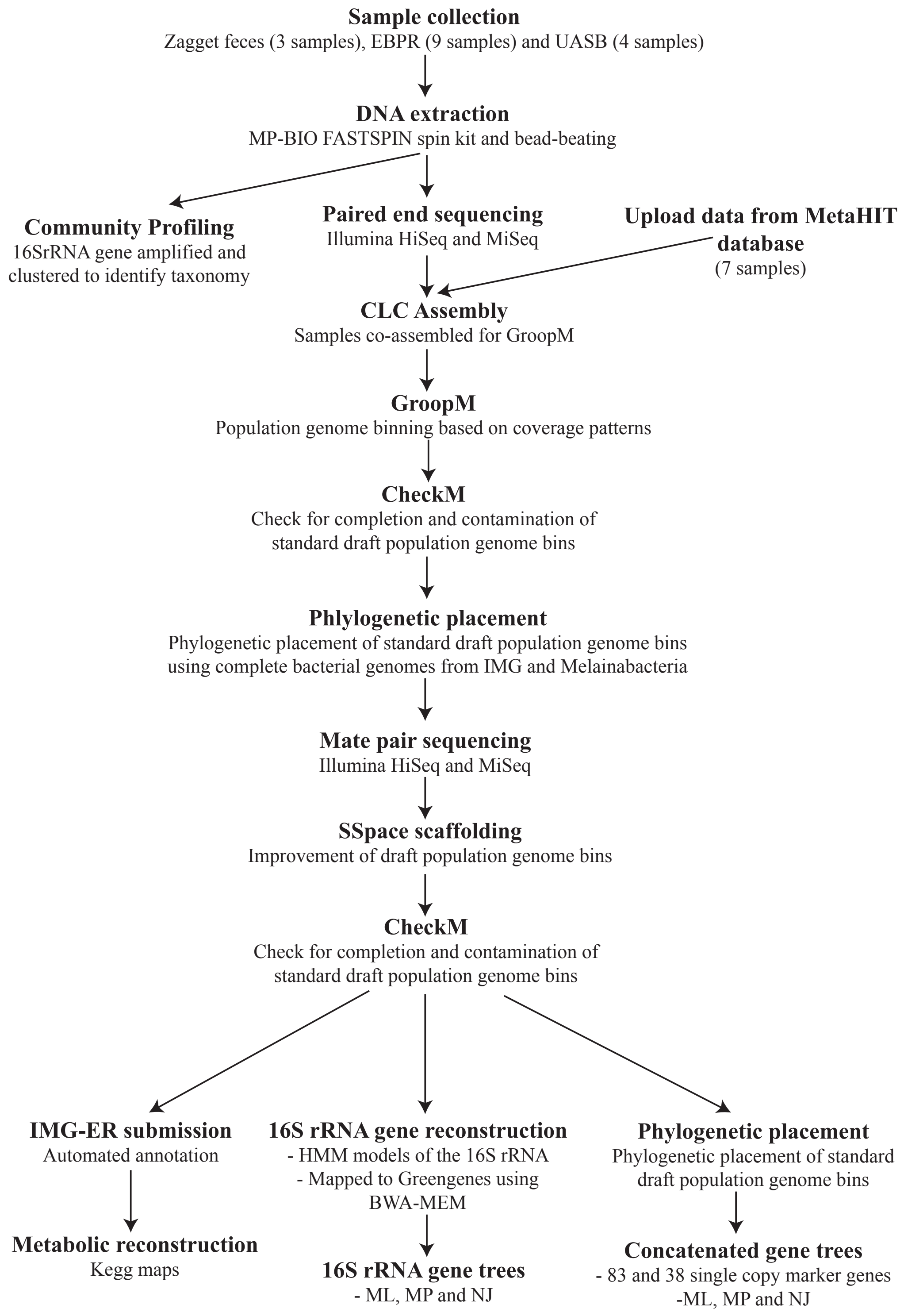

Fig. S1. Flow diagram of the Methods used in this paper

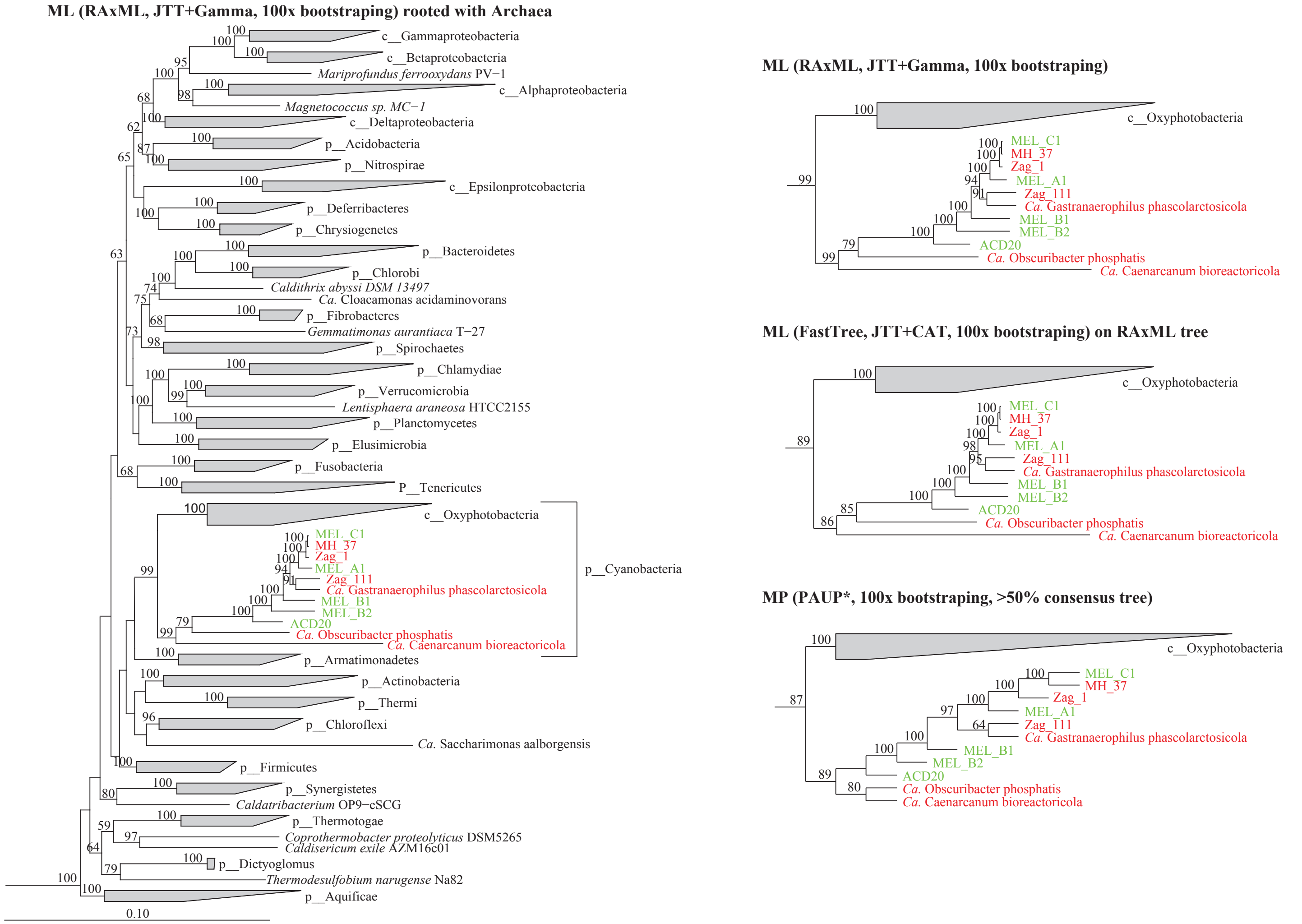

ML (RAxML, JTT+Gamma, 100x bootstraping) rooted with Archaea
c__Gammaproteobacteria
c__Betaproteobacteria
Mariprofundus ferrooxydans PV−1
c__Alphaproteobacteria
Magnetococcus sp. MC−1
c__Deltaproteobacteria
p__Acidobacteria
p__Nitrospirae
c__Epsilonproteobacteria
p__Deferribacteres
p__Chrysiogenetes
p__Bacteroidetes
p__Chlorobi
Caldithrix abyssi DSM 13497
Ca. Cloacamonas acidaminovorans
p__Fibrobacteres
Gemmatimonas aurantiaca T−27
p__Spirochaetes
p__Chlamydiae
p__Verrucomicrobia
Lentisphaera araneosa HTCC2155
p__Planctomycetes
p__Elusimicrobia
p__Fusobacteria
P__Tenericutes
c__Oxyphotobacteria
MEL_C1
MH_37
Zag_1
MEL_A1
Zag_111
Ca. Gastranaerophilus phascolarctosicola
MEL_B1
MEL_B2
ACD20
Ca. Obscuribacter phosphatis
Ca. Caenarcanum bioreactoricola
p__Cyanobacteria
p__Armatimonadetes
p__Actinobacteria
p__Thermi
p__Chloroflexi
Ca. Saccharimonas aalborgensis
p__Firmicutes
p__Synergistetes
Caldatribacterium OP9−cSCG
p__Thermotogae
Coprothermobacter proteolyticus DSM5265
Caldisericum exile AZM16c01
p__Dictyoglomus
Thermodesulfobium narugense Na82
p__Aquificae
0.10
ML (RAxML, JTT+Gamma, 100x bootstraping)
c__Oxyphotobacteria
MEL_C1
MH_37
Zag_1
MEL_A1
Zag_111
Ca. Gastranaerophilus phascolarctosicola
MEL_B1
MEL_B2
ACD20
Ca. Obscuribacter phosphatis
Ca. Caenarcanum bioreactoricola
ML (FastTree, JTT+CAT, 100x bootstraping) on RAxML tree
c__Oxyphotobacteria
MEL_C1
MH_37
Zag_1
MEL_A1
Zag_111
Ca. Gastranaerophilus phascolarctosicola
MEL_B1
MEL_B2
ACD20
Ca. Obscuribacter phosphatis
Ca. Caenarcanum bioreactoricola
MP (PAUP*, 100x bootstraping, >50% consensus tree)
c__Oxyphotobacteria
MEL_C1
MH_37
Zag_1
MEL_A1
Zag_111
Ca. Gastranaerophilus phascolarctosicola
MEL_B1
MEL_B2
ACD20
Ca. Obscuribacter phosphatis
Ca. Caenarcanum bioreactoricola

Fig. S2. Phylogeny of Oxyphotobacteria and Melainabacteria genomes among the bacterial phyla based on up to 38 marker genes

Phylogenetic maximum likelihood (RAxML, JTT, G) and maximum parsimony (PAUP*) trees based on a concatenated alignment of up to 38 marker genes, showing the phylogenetic robustness of the phylum Cyanobacteria and the classes Oxyphotobacteria and Melainabacteria. 422 OTUs (operational taxonomic units) from Bacteria and Archaea were used to produce the phylogenetic tree (**Table S4**). Bootstrap analyses (100 times) were performed for the data set with maximum likelihood (RAxML, JTT, G; FastTree, JTT, CAT) and maximum parsimony (PAUP*) methods, and the values obtained are shown in respective trees, except for the values from FastTree, which are shown on the tree generated with RAxML bootstrapping. Genomes in green are representatives from Di Rienzi et al., 2013 and genomes in red are Melainabacteria from this study. Bootstrap values >60% are shown. p_ represents phylum and c_ represents class. *Candidatus* has been abbreviated to *Ca.* for the most complete genomes.

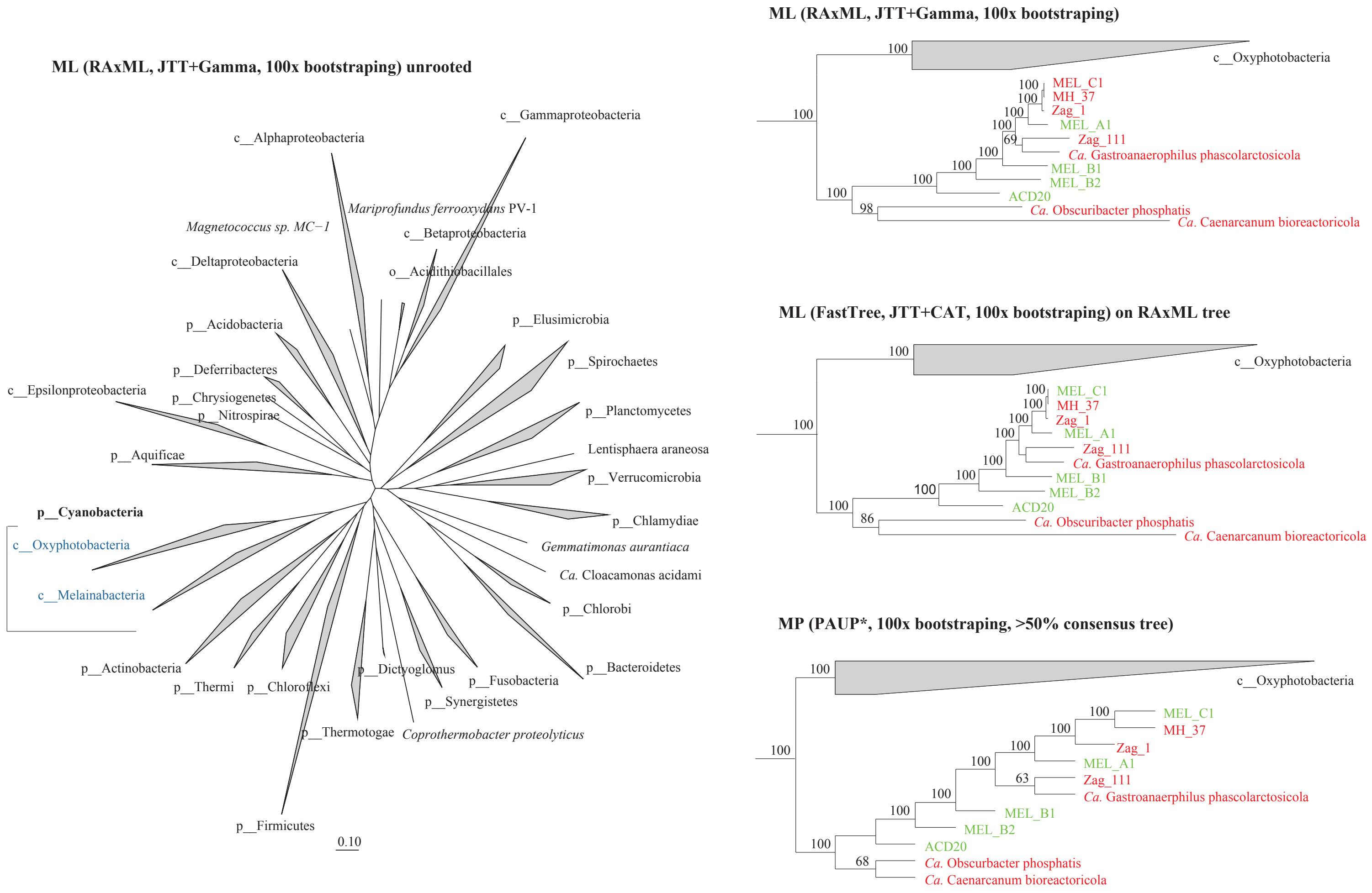

ML (RAxML, JTT+Gamma, 100x bootstraping) unrooted
c__Gammaproteobacteria
c__Alphaproteobacteria
Mariprofundus ferrooxydans PV-1
Magnetococcus sp. MC−1
c__Betaproteobacteria
c__Deltaproteobacteria
o__Acidithiobacillales
p__Acidobacteria
p__Elusimicrobia
p__Deferribacteres
p__Spirochaetes
c__Epsilonproteobacteria
p__Chrysiogenetes
p__Nitrospirae
p__Planctomycetes
Lentisphaera araneosa
p__Aquificae
p__Verrucomicrobia
p__Cyanobacteria
p__Chlamydiae
c__Oxyphotobacteria
Gemmatimonas aurantiaca
Ca. Cloacamonas acidami
c__Melainabacteria
p__Chlorobi
p__Actinobacteria
p__Bacteroidetes
p__Thermi
p__Chloroflexi
p__Dictyoglomus
p__Fusobacteria
p__Synergistetes
p__Thermotogae
Coprothermobacter proteolyticus
p__Firmicutes
0.10
ML (RAxML, JTT+Gamma, 100x bootstraping)
c__Oxyphotobacteria
MEL_C1
MH_37
Zag_1
MEL_A1
Zag_111
Ca. Gastroanaerophilus phascolarctosicola
MEL_B1
MEL_B2
ACD20
Ca. Obscuribacter phosphatis
Ca. Caenarcanum bioreactoricola
ML (FastTree, JTT+CAT, 100x bootstraping) on RAxML tree
c__Oxyphotobacteria
MEL_C1
MH_37
Zag_1
MEL_A1
Zag_111
Ca. Gastroanaerophilus phascolarctosicola
MEL_B1
MEL_B2
ACD20
Ca. Obscuribacter phosphatis
Ca. Caenarcanum bioreactoricola
MP (PAUP*, 100x bootstraping, >50% consensus tree)
c__Oxyphotobacteria
MEL_C1
MH_37
Zag_1
MEL_A1
Zag_111
Ca. Gastroanaerphilus phascolarctosicola
MEL_B1
MEL_B2
ACD20
Ca. Obscurbacter phosphatis
Ca. Caenarcanum bioreactoricola

Fig. S3. Phylogeny of Oxyphotobacteria and Melainabacteria among the bacterial phyla based on up to 83 marker genes

Phylogenetic maximum likelihood (RAxML, JTT, G) and maximum parsimony (PAUP*) trees based on a concatenated alignment of up to 83 marker genes, showing the phylogenetic robustness of the phylum Cyanobacteria and the classes Oxyphotobacteria and Melainabacteria. 322 OTUs from Bacteria were used to produce the phylogenetic tree (**Table S4**). Bootstrap analyses (100 times) were performed for the data set with maximum likelihood (RAxML, JTT, G; FastTree, JTT, CAT) and maximum parsimony (PAUP*) methods, and the values obtained are shown in respective trees, except for the values from FastTree, which are shown on the tree generated with RAxML. Genomes in green are representatives from Di Rienzi et al., 2013 and genomes in red are Melainabacteria from this study. Bootstrap values >60% are shown. p_ represents phylum, c_ represents class and o_ represents order. *Candidatus* has been abbreviated to *Ca.* for the most complete genomes.

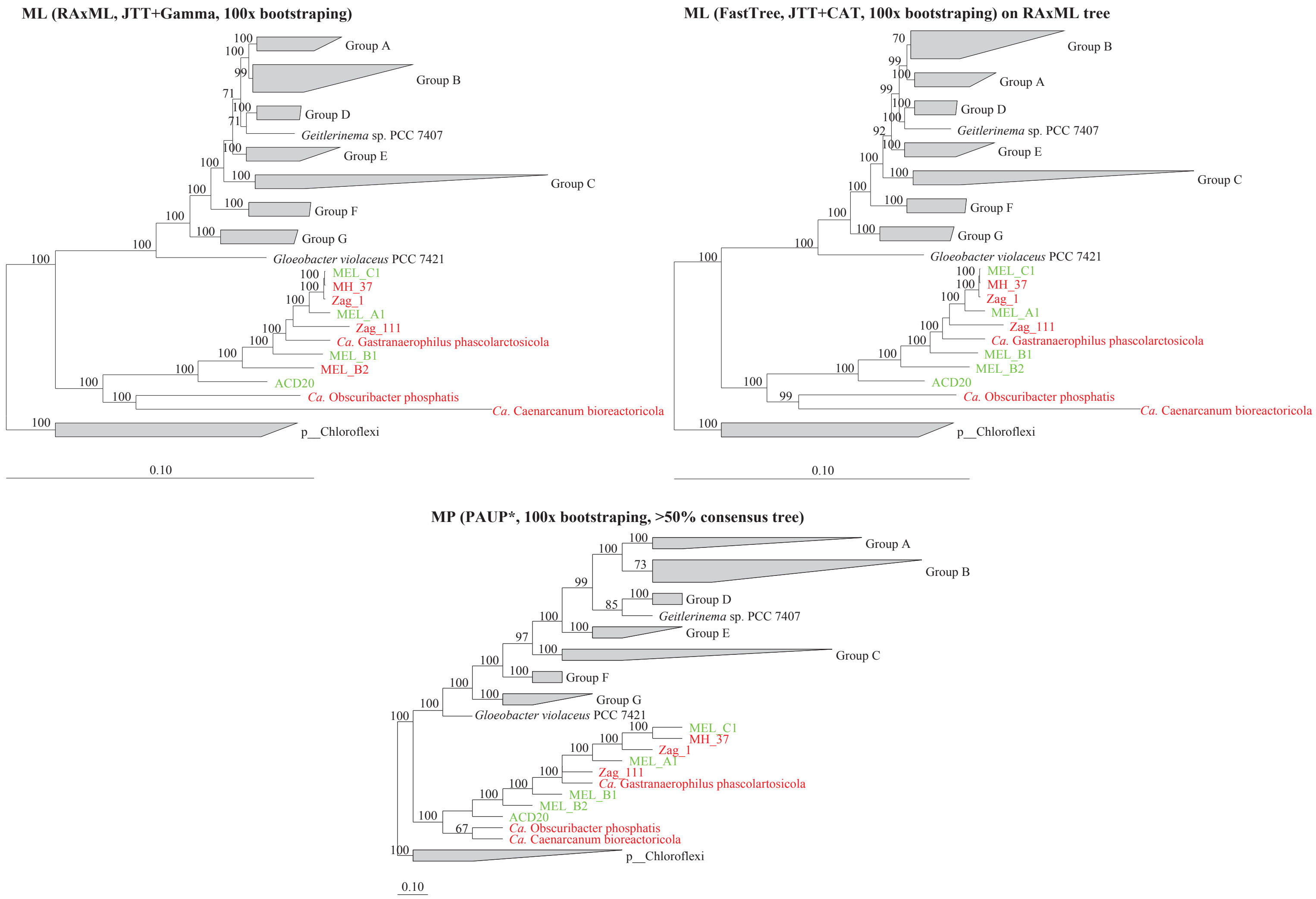
ML (RAxML, JTT+Gamma, 100x bootstraping)
Group A
Group B
Group D
Geitlerinema sp. PCC 7407
Group E
Group C
Group F
Group G
Gloeobacter violaceus PCC 7421
MEL_C1
MH_37
Zag_1
MEL_A1
Zag_111
Ca. Gastranaerophilus phascolarctosicola
MEL_B1
MEL_B2
ACD20
Ca. Obscuribacter phosphatis
Ca. Caenarcanum bioreactoricola
p__Chloroflexi
0.10
ML (FastTree, JTT+CAT, 100x bootstraping) on RAxML tree
Group B
Group A
Group D
Geitlerinema sp. PCC 7407
Group E
Group C
Group F
Group G
Gloeobacter violaceus PCC 7421
MEL_C1
MH_37
Zag_1
MEL_A1
Zag_111
Ca. Gastranaerophilus phascolarctosicola
MEL_B1
MEL_B2
ACD20
Ca. Obscuribacter phosphatis
Ca. Caenarcanum bioreactoricola
p__Chloroflexi
0.10
MP (PAUP*, 100x bootstraping, >50% consensus tree)
Group A
Group B
Group D
Geitlerinema sp. PCC 7407
Group E
Group C
Group F
Group G
Gloeobacter violaceus PCC 7421
MEL_C1
MH_37
Zag_1
MEL_A1
Zag_111
Ca. Gastranaerophilus phascolartosicola
MEL_B1
MEL_B2
ACD20
Ca. Obscuribacter phosphatis
Ca. Caenarcanum bioreactoricola
p__Chloroflexi
0.10

Fig. S4. Phylogeny of Oxyphotobacteria and Melainabacteria in the Cyanobacteria phylum based on up to 83 marker genes

Phylogenetic maximum likelihood (RAxML, JTT, G) and maximum parsimony (PAUP*) trees based on a concatenated alignment of up to 83 marker genes, showing the phylogenetic robustness of the phylum Cyanobacteria and the classes with Chloroflexi used as the outgroup. The Oxyphotobacteria were grouped as according to Shih et al., 2013 (**Table S3**). Bootstrap analyses (100 times) were performed for the data set with maximum likelihood (RAxML, JTT, G; FastTree, JTT, CAT) and maximum parsimony (PAUP*) methods, and the values obtained are shown in respective trees, except for the values from FastTree, which are shown on the tree generated with RAxML. Genomes in green are representatives from Di Rienzi et al., 2013 and genomes in red are Melainabacteria from this study. Bootstrap values >60% are shown. p_ represents phylum, c_ represents class and o_ represents order. *Candidatus* has been abbreviated to *Ca.* for the most complete genomes.

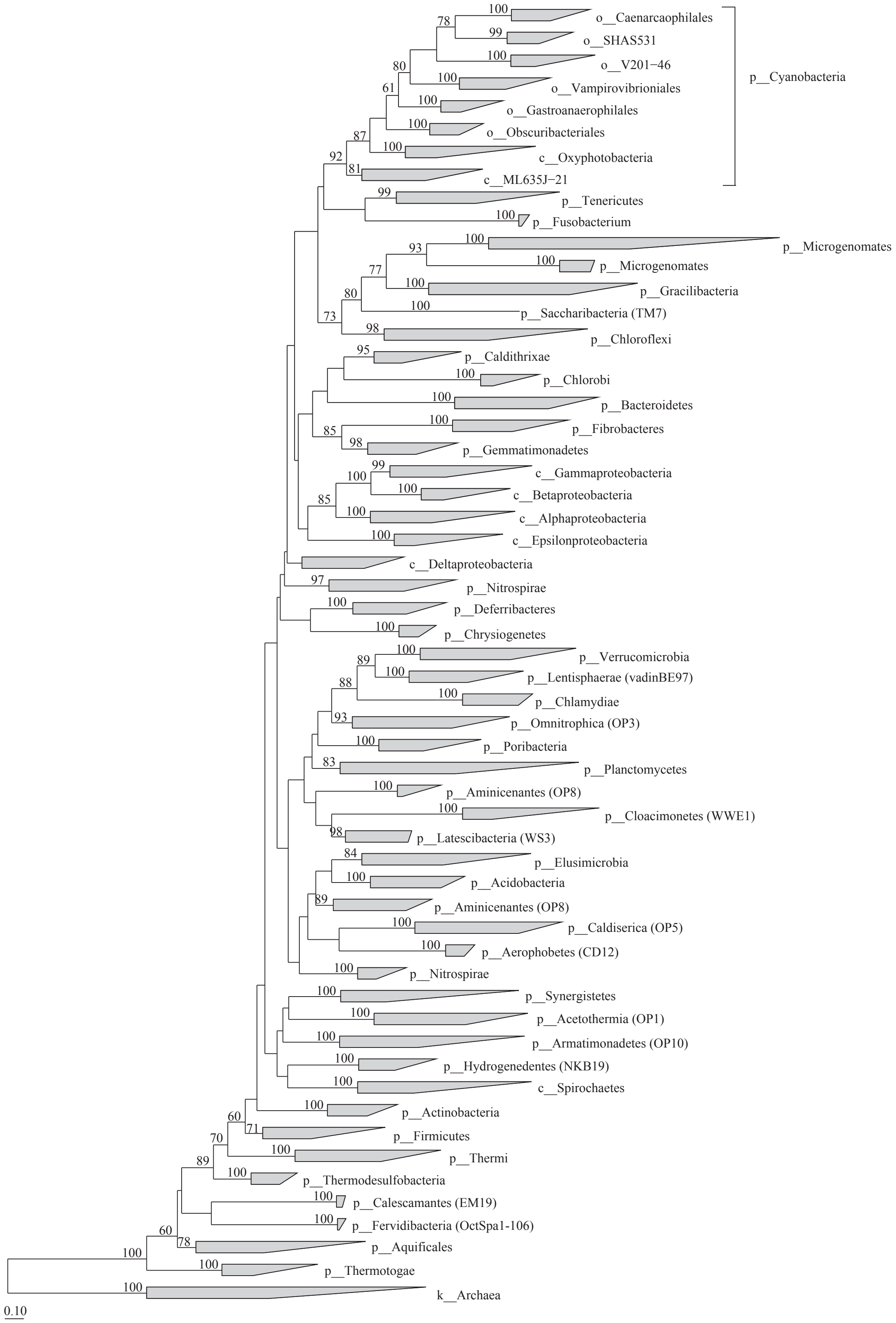
o__Caenarcaophilales
o__SHAS531
o__V201−46
o__Vampirovibrioniales
o__Gastroanaerophilales
o__Obscuribacteriales
c__Oxyphotobacteria
c__ML635J−21
p__Cyanobacteria
p__Tenericutes
p__Fusobacterium
p__Microgenomates
p__Microgenomates
p__Gracilibacteria
p__Saccharibacteria (TM7)
p__Chloroflexi
p__Caldithrixae
p__Chlorobi
p__Bacteroidetes
p__Fibrobacteres
p__Gemmatimonadetes
c__Gammaproteobacteria
c__Betaproteobacteria
c__Alphaproteobacteria
c__Epsilonproteobacteria
c__Deltaproteobacteria
p__Nitrospirae
p__Deferribacteres
p__Chrysiogenetes
p__Verrucomicrobia
p__Lentisphaerae (vadinBE97)
p__Chlamydiae
p__Omnitrophica (OP3)
p__Poribacteria
p__Planctomycetes
p__Aminicenantes (OP8)
p__Cloacimonetes (WWE1)
p__Latescibacteria (WS3)
p__Elusimicrobia
p__Acidobacteria
p__Aminicenantes (OP8)
p__Caldiserica (OP5)
p__Aerophobetes (CD12)
p__Nitrospirae
p__Synergistetes
p__Acetothermia (OP1)
p__Armatimonadetes (OP10)
p__Hydrogenedentes (NKB19)
c__Spirochaetes
p__Actinobacteria
p__Firmicutes
p__Thermi
p__Thermodesulfobacteria
p__Calescamantes (EM19)
p__Fervidibacteria (OctSpa1-106)
p__Aquificales
p__Thermotogae
k__Archaea
0.10

Fig. S5. 16S rRNA gene tree showing the phylogenetic robustness of the Cyanobacteria and its class level lineages in the bacterial domain

Maximum likelihood (RAxML, GTR, G, I; FastTree, JTT, CAT) trees based on the 16S rRNA gene, showing the phylogenetic robustness of the classes Oxyphotobacteria and Melainabacteria. 418 OTUs (operational taxonomic units) from Bacteria and Archaea were used to construct the trees. Bootstrap analyses (100 times) were performed for the data set with maximum likelihood (RAxML, GTR, G, I; FastTree, JTT, CAT). Bootstrap values >60% are shown.

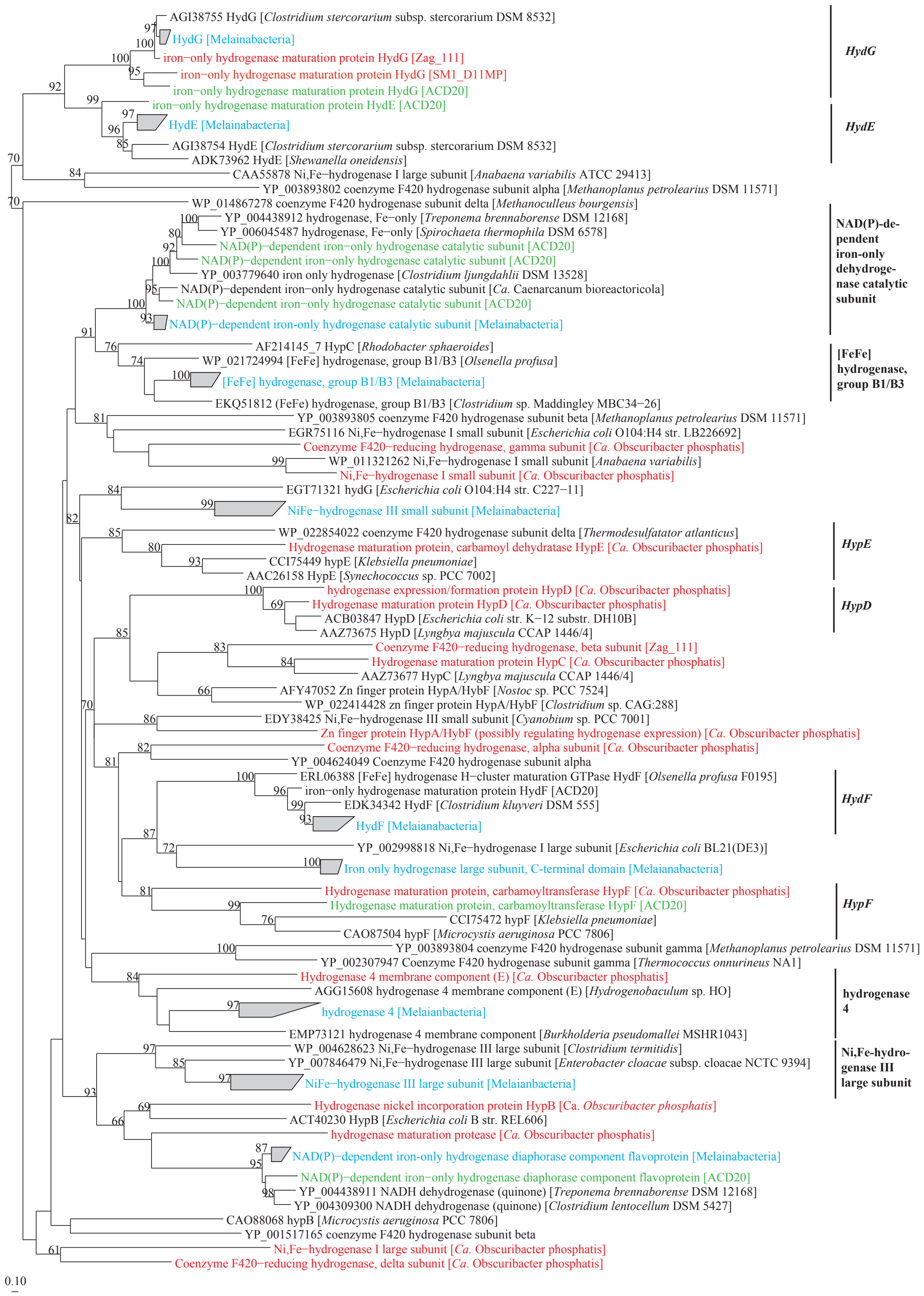
AGI38755 HydG [Clostridium stercorarium subsp. stercorarium DSM 8532]
HydG [Melainabacteria]
iron−only hydrogenase maturation protein HydG [Zag_111]
iron−only hydrogenase maturation protein HydG [SM1_D11MP]
iron−only hydrogenase maturation protein HydG [ACD20]
iron−only hydrogenase maturation protein HydE [ACD20]
HydE [Melainabacteria]
AGI38754 HydE [Clostridium stercorarium subsp. stercorarium DSM 8532]
ADK73962 HydE [Shewanella oneidensis]
CAA55878 Ni,Fe−hydrogenase I large subunit [Anabaena variabilis ATCC 29413]
YP_003893802 coenzyme F420 hydrogenase subunit alpha [Methanoplanus petrolearius DSM 11571]
WP_014867278 coenzyme F420 hydrogenase subunit delta [Methanoculleus bourgensis]
YP_004438912 hydrogenase, Fe−only [Treponema brennaborense DSM 12168]
YP_006045487 hydrogenase, Fe−only [Spirochaeta thermophila DSM 6578]
NAD(P)−dependent iron−only hydrogenase catalytic subunit [ACD20]
NAD(P)−dependent iron−only hydrogenase catalytic subunit [ACD20]
YP_003779640 iron only hydrogenase [Clostridium ljungdahlii DSM 13528]
NAD(P)−dependent iron−only hydrogenase catalytic subunit [Ca. Caenarcanum bioreactoricola]
NAD(P)−dependent iron−only hydrogenase catalytic subunit [ACD20]
NAD(P)−dependent iron-only hydrogenase catalytic subunit [Melainabacteria]
AF214145_7 HypC [Rhodobacter sphaeroides]
WP_021724994 [FeFe] hydrogenase, group B1/B3 [Olsenella profusa]
[FeFe] hydrogenase, group B1/B3 [Melainabacteria]
EKQ51812 (FeFe) hydrogenase, group B1/B3 [Clostridium sp. Maddingley MBC34−26]
YP_003893805 coenzyme F420 hydrogenase subunit beta [Methanoplanus petrolearius DSM 11571]
EGR75116 Ni,Fe−hydrogenase I small subunit [Escherichia coli O104:H4 str. LB226692]
Coenzyme F420−reducing hydrogenase, gamma subunit [Ca. Obscuribacter phosphatis]
WP_011321262 Ni,Fe−hydrogenase I small subunit [Anabaena variabilis]
Ni,Fe−hydrogenase I small subunit [Ca. Obscuribacter phosphatis]
EGT71321 hydG [Escherichia coli O104:H4 str. C227−11]
NiFe−hydrogenase III small subunit [Melainabacteria]
WP_022854022 coenzyme F420 hydrogenase subunit delta [Thermodesulfatator atlanticus]
Hydrogenase maturation protein, carbamoyl dehydratase HypE [Ca. Obscuribacter phosphatis]
CCI75449 hypE [Klebsiella pneumoniae]
AAC26158 HypE [Synechococcus sp. PCC 7002]
hydrogenase expression/formation protein HypD [Ca. Obscuribacter phosphatis]
Hydrogenase maturation protein HypD [Ca. Obscuribacter phosphatis]
ACB03847 HypD [Escherichia coli str. K−12 substr. DH10B]
AAZ73675 HypD [Lyngbya majuscula CCAP 1446/4]
Coenzyme F420−reducing hydrogenase, beta subunit [Zag_111]
Hydrogenase maturation protein HypC [Ca. Obscuribacter phosphatis]
AAZ73677 HypC [Lyngbya majuscula CCAP 1446/4]
AFY47052 Zn finger protein HypA/HybF [Nostoc sp. PCC 7524]
WP_022414428 zn finger protein HypA/HybF [Clostridium sp. CAG:288]
EDY38425 Ni,Fe−hydrogenase III small subunit [Cyanobium sp. PCC 7001]
Zn finger protein HypA/HybF (possibly regulating hydrogenase expression) [Ca. Obscuribacter phosphatis]
Coenzyme F420−reducing hydrogenase, alpha subunit [Ca. Obscuribacter phosphatis]
YP_004624049 Coenzyme F420 hydrogenase subunit alpha
ERL06388 [FeFe] hydrogenase H−cluster maturation GTPase HydF [Olsenella profusa F0195]
iron−only hydrogenase maturation protein HydF [ACD20]
EDK34342 HydF [Clostridium kluyveri DSM 555]
HydF [Melaianabacteria]
YP_002998818 Ni,Fe−hydrogenase I large subunit [Escherichia coli BL21(DE3)]
Iron only hydrogenase large subunit, C-terminal domain [Melaianabacteria]
Hydrogenase maturation protein, carbamoyltransferase HypF [Ca. Obscuribacter phosphatis]
Hydrogenase maturation protein, carbamoyltransferase HypF [ACD20]
CCI75472 hypF [Klebsiella pneumoniae]
CAO87504 hypF [Microcystis aeruginosa PCC 7806]
YP_003893804 coenzyme F420 hydrogenase subunit gamma [Methanoplanus petrolearius DSM 11571]
YP_002307947 Coenzyme F420 hydrogenase subunit gamma [Thermococcus onnurineus NA1]
Hydrogenase 4 membrane component (E) [Ca. Obscuribacter phosphatis]
AGG15608 hydrogenase 4 membrane component (E) [Hydrogenobaculum sp. HO]
hydrogenase 4 [Melaianbacteria]
EMP73121 hydrogenase 4 membrane component [Burkholderia pseudomallei MSHR1043]
WP_004628623 Ni,Fe−hydrogenase III large subunit [Clostridium termitidis]
YP_007846479 Ni,Fe−hydrogenase III large subunit [Enterobacter cloacae subsp. cloacae NCTC 9394]
NiFe−hydrogenase III large subunit [Melaianbacteria]
Hydrogenase nickel incorporation protein HypB [Ca. Obscuribacter phosphatis]
ACT40230 HypB [Escherichia coli B str. REL606]
hydrogenase maturation protease [Ca. Obscuribacter phosphatis]
NAD(P)−dependent iron-only hydrogenase diaphorase component flavoprotein [Melainabacteria]
NAD(P)−dependent iron−only hydrogenase diaphorase component flavoprotein [ACD20]
YP_004438911 NADH dehydrogenase (quinone) [Treponema brennaborense DSM 12168]
YP_004309300 NADH dehydrogenase (quinone) [Clostridium lentocellum DSM 5427]
CAO88068 hypB [Microcystis aeruginosa PCC 7806]
YP_001517165 coenzyme F420 hydrogenase subunit beta
Ni,Fe−hydrogenase I large subunit [Ca. Obscuribacter phosphatis]
Coenzyme F420−reducing hydrogenase, delta subunit [Ca. Obscuribacter phosphatis]
HydG
HydE
NAD(P)-dependent iron-only dehydrogenase catalytic subunit
[FeFe] hydrogenase, group B1/B3
HypE
HypD
HydF
HypF
hydrogenase 4
Ni,Fe-hydrogenase III large subunit
0.10

Fig. S6. Gene tree showing the hydrogenase genes found in the Melainabacteria representatives.

A representative collection of hydrogenase genes publicly available from NCBI was combined with genes identified from this study and from Di Rienzi et al., 2013. Hydrogenase genes common to genomes from both studies are colored blue and grouped together. Hydrogenases found only in this study are colored red and genes only identified in the genomes of Di Rienzi et al., 2013 are colored green.

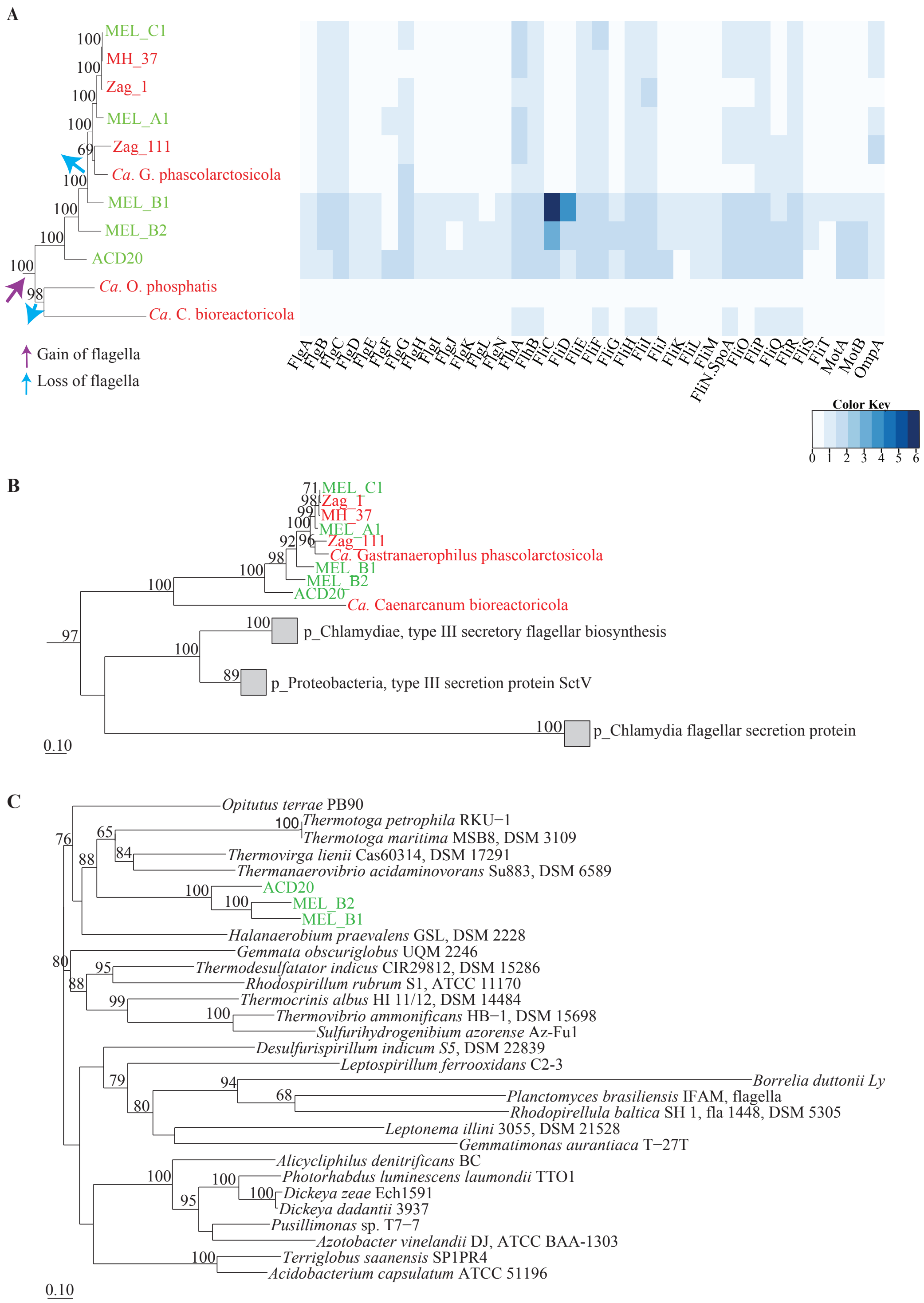

A
MEL_C1
MH_37
Zag_1
MEL_A1
Zag_111
Ca. G. phascolarctosicola
MEL_B1
MEL_B2
ACD20
Ca. O. phosphatis
Ca. C. bioreactoricola
100
100
100
100
69
100
100
100
100
98
Gain of flagella
Loss of flagella
FlgA FlgB FlgC FlgD FlgE FlgF FlgG FlgH FlgI FlgJ FlgK FlgL FlgN FlhA FlhB FliC FliD FliE FliF FliG FliH FliI FliJ FliK FliL FliM FliN.SpoA FliO FliP FliQ FliR FliS FliT MotA MotB OmpA
Color Key
0 1 2 3 4 5 6
B
71 MEL_C1
98 Zag_1
99 MH_37
100 MEL_A1
92 96 Zag_111
Ca. Gastranaerophilus phascolarctosicola
98 MEL_B1
100 MEL_B2
100 ACD20
Ca. Caenarcanum bioreactoricola
97
100 p_Chlamydiae, type III secretory flagellar biosynthesis
100
89 p_Proteobacteria, type III secretion protein SctV
100 p_Chlamydia flagellar secretion protein
0.10
C
Opitutus terrae PB90
100 Thermotoga petrophila RKU−1
Thermotoga maritima MSB8, DSM 3109
Thermovirga lienii Cas60314, DSM 17291
Thermanaerovibrio acidaminovorans Su883, DSM 6589
100 ACD20
100 MEL_B2
MEL_B1
Halanaerobium praevalens GSL, DSM 2228
Gemmata obscuriglobus UQM 2246
95 Thermodesulfatator indicus CIR29812, DSM 15286
Rhodospirillum rubrum S1, ATCC 11170
99 Thermocrinis albus HI 11/12, DSM 14484
100 Thermovibrio ammonificans HB−1, DSM 15698
Sulfurihydrogenibium azorense Az-Fu1
Desulfurispirillum indicum S5, DSM 22839
Leptospirillum ferrooxidans C2-3
94 Borrelia duttonii Ly
68 Planctomyces brasiliensis IFAM, flagella
Rhodopirellula baltica SH 1, fla 1448, DSM 5305
79
80
Leptonema illini 3055, DSM 21528
Gemmatimonas aurantiaca T−27T
Alicycliphilus denitrificans BC
100
100 Photorhabdus luminescens laumondii TTO1
100 Dickeya zeae Ech1591
95 Dickeya dadantii 3937
Pusillimonas sp. T7−7
Azotobacter vinelandii DJ, ATCC BAA-1303
100 Terriglobus saanensis SP1PR4
Acidobacterium capsulatum ATCC 51196
76
65
84
88
80
88
0.10

Fig. S7. Heat map showing the presence and absence of flagella genes from the Melainabacteria representatives

(A) A maximum parsimony tree based on a concatenated alignment of up to 83 marker genes. Purple arrows indicate the gain of a functional flagella and blue arrows indicate the loss of a functional flagella. The heat map indicates the square root of the number of flagella genes found in each of the Melainabacteria representatives. (B) Flagella gene trees for FlhA and (C) flagella gene tree for FliM. Genomes used to make the trees are found in **Table S6**. Genomes in green are Melainabacteria representatives from Di Rienzi et al., 2013, genomes in red are Melaianabacteria representatives from this study. *Candidatus* has been abbreviated to *Ca.* for the most complete genomes.

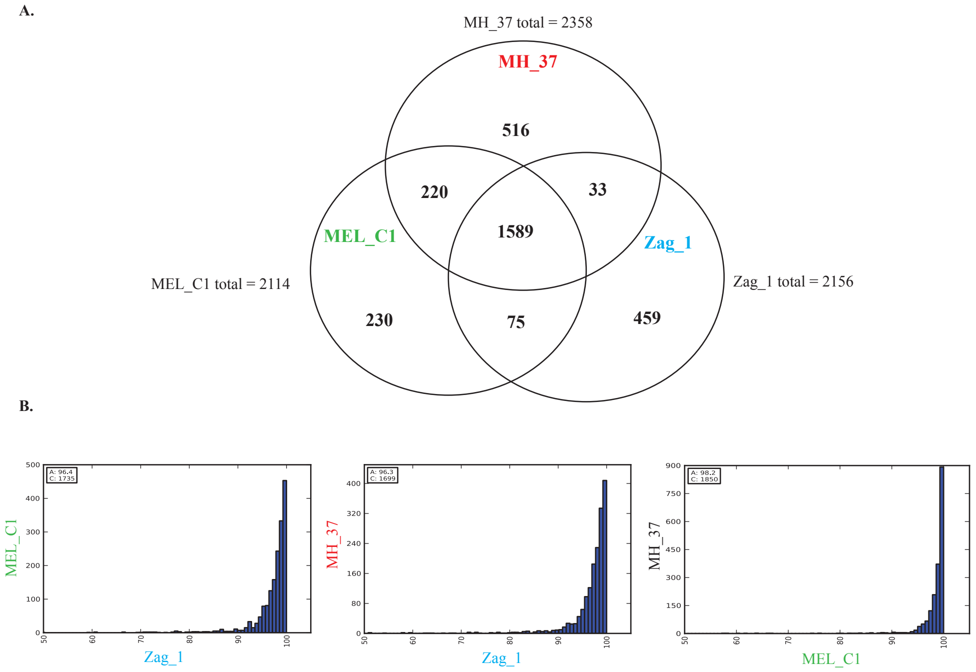

Fig. S8. Venn diagram of the three Gastranaerophilales genomes from the same species

(A) Shared orthologs between MH_37, MEL_C1 and Zag_1 showing the number of genes that are core to the three genomes, and those that are found between two genomes and individual genomes. The total number for each genome does not include paralogs. (B) Average nucleotide identity between MH_37, MEL_C1 and Zag_1, where ≥95% ANI is used to define a species.

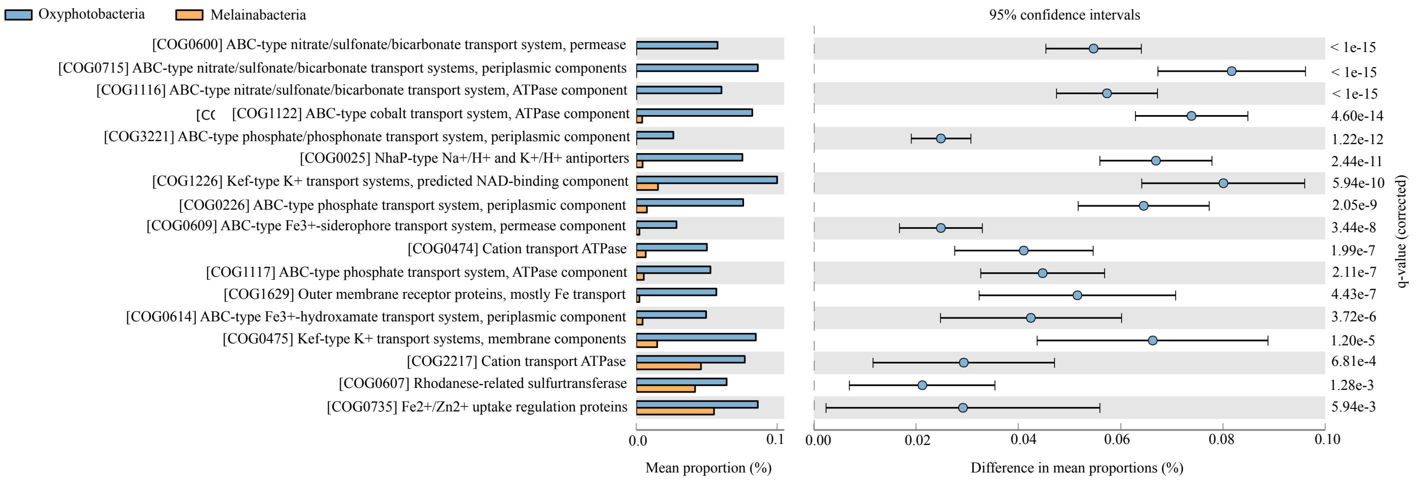

Fig. S9. COG category P (inorganic ion transport and metabolism) for Oxyphotobacteria and Melainabacteria

Shown are all COGs from category P with a difference in mean proportions >= 0.02% between Oxyphotobacteria and Melainabacteria and a Storey's q-value of <= 0.05.

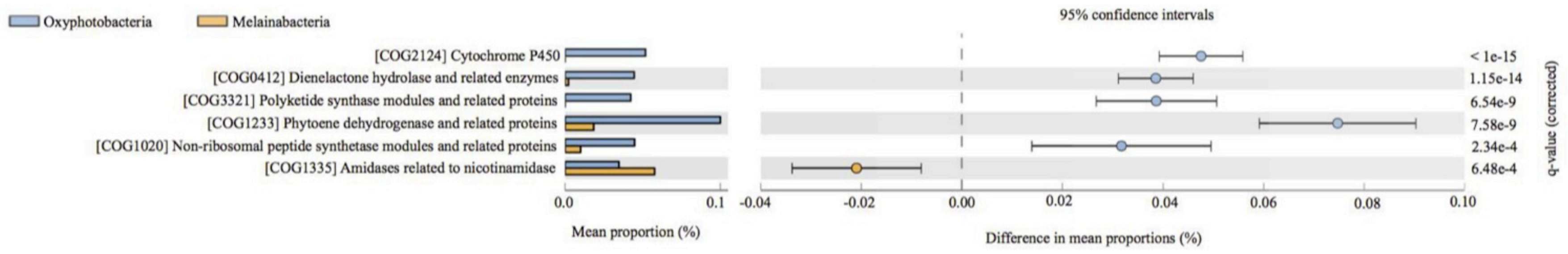

Fig. S10. COG category Q (secondary metabolites and biosynthesis, transport and catabolism) for Oxyphotobacteria and Melainabacteria

Shown are all COGs from category Q with a difference in mean proportions >= 0.02% between Oxyphotobacteria and Melainabacteria and a Storey's q-value of <= 0.05
