# Supplementary material for: An Expanded Genomic Representation of the Phylum Cyanobacteria
Source: Genome Biol Evol. 2014 May 2;6(5):1031–45. doi: 10.1093/gbe/evu073 (PMC4040986; doi:10.1093/gbe/evu073)
Supplement: Supplementary Data [file supp_evu073_Soo_GBE_Supplementary_Tables.pdf]

Supplementary Tables for

An Expanded Genomic Representation of the Phylum Cyanobacteria

Rochelle M. Soo, Connor T. Skennerton, Yuji Sekiguchi, Michael Imelfort, Samuel J. Paech, Paul G. Dennis, Jason A. Steen, Donovan H. Parks, Gene W. Tyson, and Philip Hugenholtz[†]

[†] Correspondence to: Philip Hugenholtz, p.hugenholtz@uq.edu.au

Table S1. Sequencing statistics

EBPR1_T1 to EBPR1_T6 and EBPR2_T1 to EBPR2_T3 (blue) correspond to the nine samples collected from two enhanced biological phosphorous removal bioreactor (EBPR), Zag_T1 to Zag_T3 (red) correspond to the three time points where samples were collected from koala feces, MH_F2, F3, F5, F6, F8, M3 and M8 correspond to human feces collected from seven Danish females and males (http://www.metahit.eu/) (purple) and A1, A2, F1 and F2 from the UASB (green). The combined assembly statistics is the amount of sequencing performed for all of the samples collected from EBPR, koala feces, Danish individuals or UASB. Genome population bins is the number of genome bins that was produced by GroopM v.1.0. The N50 is for all of the combined metagenomic data for each sample.

		Combined shotgun sequencing assembly statistics for GroopM				Mate pair sequencing	
**Sample ID**	**Sampling Date**	**Shotgun sequence (Gbp)**	**Number of contigs**	**N50**	**Genome population bins**	**Mate pair sequencing (Gbp)**	**Insert size (kbp)**
EBPR1_T1	05/27/11	26.2 (174,720,232 x 150bp)	148,338	1.4 kbp	299	4.88	3.2-3.8
EBPR1_T2	06/22/11	21.49 (143,317,626 x 150bp)					
EBPR1_T3	08/01/11	23.76 (158,451,996 x 150bp)					
EBPR1_T4	09/08/11	38.01 (253,461,380 x 150bp)					
EBPR1_T5	11/25/11	16.78 (111,886,692 x 150bp)					
EBPR1_T6	01/18/12	22.65 (151,023,810 x 150bp)				5.72	3.2-3.8
EBPR2_T1	06/17/11	18.86 (125,756,890 x 150bp)				6.15	3.2-3.8
EBPR2_T2	09/05/11	19.08 (127,217,526 x 150bp)					
EBPR2_T3	12/16/11	24.81 (165,419,806 x 150bp)				5.10	3.2-3.8
Zag_T1	05/12/11	10.68 (71,196,258 x 150bp)	17,101	4.6 kbp	181	1.99	2-15
Zag_T2	07/28/11	65.06 (433,763,472 x 150bp)				1.89	2-15
Zag_T3	11/24/11	15.00 (100,022,578 x 150bp)				1.87	2-15
UASB_A1	12/25/12	7.3 (32,365,294 x 250bp)	84,262	2.7 kbp	154	2.00	5.5

UASB_A2	09/16/10	5.8 (23,420,132 x 250bp)						
UASB_F1	-	5.0 (23,027,350 x 250bp)						
UASB_G1	-	7.8 (35,467,130 x 250bp)						
MH_F2 (M0002)	-	3.49 (46,574,230 x 75bp)	3,139	2.3 kbp	119			
MH_F3 (MH0006)	-	6.96 (92,764,998 x 75bp)						
MH_F5 (MH0021)	-	1.97 (26,262,536 x 75bp)						
MH_F6 (MH0024)	-	1.61 (21,421,864 x 75bp)						
MH_F8 (MH0028)	-	1.55 (20,637,196 x 75bp)						
MH_M3 (MH0009)	-	4.38 (58,458,876 x 75bp)						
MH_M8 (MH0031)	-	1.62 (21,566,850 x 75bp)						

Table S2. List of 83 single copy gene markers

The list of 83 single copy gene markers is a subset of the 111 single copy gene markers compiled by Dupont et al., 2012.

TIGR/PFAM	Name	Size (aa)
TIGR00064	ftsY: Signal recognition particle-docking protein FtsY	279
TIGR00082	rbfA: ribosome-binding factor A	115
TIGR00086	smpB: SsrA-binding protein	144
TIRG00092	TIGR00092: GTP-binding protein YchF	368
TIGR00115	tig: trigger factor	410
TIGR00116	tsf: translation elongation factor Ts	293
TIGR00158	L9: ribosomal protein L9	148
TIGR00165	S18: ribosomal protein S18	70
TIGR00337	PyrG: CTP synthase	526
TIGR00344	alaS: alanine—tRNA ligase	847
TIGR02386	rpoC_TIGR: DNA-directed RNA polymerase, beta subunit	1147
TIGR02387	rpoC1_cyan: DNA-directed RNA polymerase, gamma subunit	619
TIGR02397	dnaX_nterm: DNA polymerase III, subunit gamma and tau	355
TIGR02729	Obg_CgtA: Obg family GTPase CgtA	329
TIGR02012	tigrfam_recA: protein RecA	321
TIGR02013	rpoB: DNA-directed RNA polymerase, beta subunit	1238
TIGR02027	rpoA: DNA-directed RNA polymerase, alpha subunit	298
TIGR03263	guanyl_kin: guanylate kinase	180
TIGR03594	GTPase_EngA: ribosome-associated GTPase EngA	432
TIGR00409	proS_fam_II: proline—tRNA ligase	568
PF00162	Phosphoglycerate kinase	384
PF00276	Ribosomal protein L23	92
PF00281	Ribosomal protein L5	56
PF00297	Ribosomal protein L3	263
PF00380	Ribosomal protein S9/S16	121
PF00410	Ribosomal protein S8	129
PF00411	Ribosomal protein S11	110
PF00416	Ribosomal protein S13/S18	106
PF00466	Ribosomal protein L10	100
PF00573	Ribosomal protein L4/L1 family	192
PF01795	MraW methylase family	310
TIGR00001	rpml_bact: ribosomal protein L35	63
TIGR00002	S16: ribosomal protein S16	78
TIGR00019	prfA: peptide chain release factor 1	361
TIGR00029	S20: ribosomal protein S20	87
TIGR00043	TIGR00043: metalloprotein, YbeY/UPF0054 family	111
TIGR00059	L17: ribosomal protein L17	112
TIGR00060	L18_bact: ribosomal protein L18	114
TIGR00061	L21: ribosomal protein L21	101
TIGR00166	S6: ribosomal protein S6	95
TIGR00168	infC: translation initiation factor IF-3	165
TIGR00362	DnaA: chromosomal replication initiator protein DnaA	437
TIGR00388	glyQ: glycine—tRNA ligase, alpha subunit	293

TIGR00389	glyS_dimeric: glycine—tRNA ligase	565
TIGR00459	aspS_bact: aspartate—tRNA ligase	586
TIGR00460	fmt: methionyl-tRNA formyltransferase	315
TIGR00468	pheS: phenylalainin—tRNA ligase, alpha subunit	324
TIGR00959	ffh: signal recognition particle protein	428
TIGR00963	secA: preprotein translocase, SecA subunit	787
TIGR00964	secE_bact: preprotein translocase, SecE subunit	57
TIGR00967	3a0501s007: preprotein translocase, SecY subunit	414
TIGR00981	rpsL_bact: ribosomal protein S12	124
TIGR01009	rpsC_bact: ribosomal protein S3	212
TIGR01011	rpsB_bact: ribosomal protein S2	225
TIGR01021	rpsE_bact: ribosomal protein S5	156
TIGR01024	rplS_bact: ribosomal protein L19	114
TIGR01029	rpsG_bact: ribosomal protein S7	154
TIGR01032	rplT_bact: ribosomal protein L20	114
TIGR01044	rplV_bact: ribosomal protein L22	103
TIGR01049	rpsJ_bact: ribosomal protein S10	99
TIGR01050	rpsS_bact: ribosomal protein S19	92
TIGR01063	gyrA: DNA gyrase, A subunit	800
TIGR01066	rplM_bact: ribosomal protein L13	141
TIGR01067	rplN_bact: ribosomal protein L14	122
TIGR01071	rplO_bact: ribosomal protein L15	144
TIGR01079	rplX_bact: ribosomal protein L24	104
TIGR00471	pheT_arch: phenylalanine—tRNA ligase, beta subunit	551
TIGR00472	pheT_bact: phenylalanine—tRNA ligase, beta subunit	798
TIGR00487	IF-2: translation initiation factor IF-2	587
TIGR00496	frr: ribosome recycling factor	176
TIGR00575	dnlj: DNA ligase, NAD-dependent	652
TIGR00631	uvrb: excinuclease ABC subunit B	658
TIGR00663	dnan: DNA polymerase III, beta subunit	367
TIGR00810	secG: preprotein translocase, SecG subunit	73
TIGR00855	L12: ribosomal protein L7/L12	125
TIGR00922	nusG: transcription termination/antitermination factor NusG	172
TIGR01164	rplP_bact: ribosomal protein L16	126
TIGR01169	rplA_bact: ribosomal protein L1	227
TIGR01171	rplB_bact: ribosomal protein L2	275
TIGR01391	dnaG: DNA primase	414
TIGR01393	lepA: GTP-binding protein LepA	595
TIGR01632	L11_bact: ribosomal protein L11	140
TIGR01953	NusA: transcription termination factor NusA	340

Table S3. List of Oxyphotobacteria, Melainabacteria and Chloroflexi Oxyphotobacteria representatives from IMG, as grouped by Shih et al., 2013 (group A to group G), Melainabacteria from Di Rienzi et al., 2013 and this study, as well as Chloroflexi from JGI IMG that were used to make the concatenated gene tree (**Fig. 1A**).

Phylum Cyanobacteria	
Class Oxyphotobacteria	**IMG Accession**
**Group A**	
Arthrospira maxima CS-328	642979357
Arthrospira platensis C1	2507262036
Arthrospira sp. PCC 8005	648276619
Arthrospira platensis NIES-39	650377906
Lyngbya sp. CCY 8106	639857035
Trichodesmium erythraeum IMS101	637000329
Oscillatoria sp. PCC 6506	648276706
Oscillatoria nigro-viridis PCC 7112	2503982035
Oscillatoria acuminata PCC 6304	2509276028
**Group B**	
Cyanothece sp. BH68, ATCC 51142	641522622
Crocosphaera watsonii WH 8501	2531839001
Cyanobacterium UCYN-A	646311970
Cyanothece sp. PCC 8801	643348535
Synechocystis sp. PCC 6803	637000315
Cyanothece sp. PCC 7424	643348533
Cyanothece sp. PCC 7822	648028021
Microcystis aeruginosa NIES-843	641522640
Pleurocapsa sp. PCC 7327	2509276061
Synechococcus sp. PCC 7002	641522654
Leptolyngbya sp. PCC 7376	2503754048
Cyanobacterium stanieri PCC 7202	2503283023
Cyanobacterium aponinum PCC 10605	2503707009
Stanieria cyanosphaera PCC 7437	2503754019
Chroococcidiopsis sp. PCC 6712	2505679029
Spirulina major PCC 6313	2506520014
Spirulina subsalsa PCC 9445	2506520011
Dactylococcopsis salina PCC 8305	2509276056
Halothece sp. PCC 7418	2503538028
Microcoleus chthonoplastes PCC 7420	647533184
Microcoleus sp. PCC 7113	2509276031
Cylindrospermopsis raciborskii CS-505	647000233
Raphidiopsis brookii D9	647000303
Nostoc azollae 0708	648028001
Anabaena cylindrica PCC 7122	2503982047
Anabaena sp. PCC 7108	2506485002
Nostoc punctiforme PCC 73102	642555144
Calothrix sp. PCC 7507	2505679032

Nodularia spumigena CCY9414	639857037
Nostoc sp. PCC 7120	637000199
Anabaena variabilis ATCC 29413	646564504
Nostoc sp. PCC 7524	2509601032
Nostoc sp. PCC 7107	2503707008
Rivularia sp. PCC 7116	2510065008
Calothrix sp. PCC 6303	2503982036
Fischerella sp. JSC-11	2505679024
Gloeocapsa sp. PCC 7428	2503754017
Chroococcidiopsis thermalis PCC 7203	2503538021
Crinalium epipsammum PCC 9333	2504643013
**Group C**	
Synechococcus sp. CC9616	2517093019
Prochlorococcus sp. WH8102	637000314
Prochlorococcus sp. CC9605	637000310
Prochlorococcus sp. CC9902	637000311
Prochlorococcus sp. CC9311	637000309
Synechococcus sp. WH 8016	2507262052
Prochlorococcus sp. WH 7803	640427149
Prochlorococcus marinus pastoris CCMP 1986	637000214
Prochlorococcus marinus MIT 9515	640069324
Prochlorococcus marinus AS9601	640069321
Prochlorococcus marinus NATL2A	637000212
Prochlorococcus marinus marinus CCMP 1375	637000213
Prochlorococcus marinus MIT 9211	641228501
Prochlorococcus marinus MIT 9313	637000211
Cyanobium sp. PCC 7001	647533126
Cyanobium gracile PCC 6307	2508501011
Synechococcus sp. RCC307	640427148
Synechococcus elongatus PCC 6301	637000307
Synechococcus elongatus PCC 7942	637000308
**Group D**	
Cyanobacterium sp. JSC-1	2502171143
Oscillatoriales sp. JSC-12	2510065010
**Group E**	
Thermosynechococcus elongatus BP-1	637000320
Synechococcus sp. PCC 6312	2509276030
Cyanothece sp. PCC 7425	643348534
Acaryochloris marina MBIC11017	641228474
**Group F**	
Pseudanabaena sp. PCC 7367	2504643012
Synechococcus sp. PCC 7502	2508501041
**Group G**	
Synechococcus sp. JA-3-3Ab	637000313
Synechococcus sp. PE A4 65AY6A	2512875021
Synechococcus sp. JA-2-3B	637000312
Synechococcus sp. PCC 7336	2506520048

Gloeobacter violaceus PCC 7421	637000121
Geitlerinema sp. PCC 7407	2503538020
**Class Melainabacteria**	
Ca. Obscuribacter phosphatis	2541046960
Ca. Caenarcanophila bioreactus	2531839742
Ca. Gastroanaerophila phascolarctos	2523533519
Za_1	2523533517
Zag_111	2531839741
MH_37	2522572068
ACD20	2541046958
MEL_A1	2541016959
MEL_B1	2541046956
MEL_B2	2541046940
MEL_C1	2541046938
Phylum Chloroflexi	
Class Chloroflexi	
Chloroflexus aurantiacus J-10-fl	641228485
Chloroflexus aggregans DSM 9485	643348527
Oscillochloris trichoides DG6	649989977
Roseiflexus sp. RS-1	640427139
Roseiflexus castenholzii HLO8, DSM 13941	640753047
Herpetosiphon aurantiacus DSM 785	2508501111
**Class Thermomicrobia**	
Thermomicrobium roseum DSM 5159	643348582
Sphaerobacter terhmophilus 4ac11, DSM 20745	646311953
Thermobaculum terrenum YNP1 ATCC BAA-798	646311962
**Class Anaerolineae**	
Anaerolinea thermophila UNI-1	649633005
**Class Dehalococcoidia**	
Dehalococcoides sp. BAV1	640427111
Dehalogenimonas lykanthroporepellens BL-DC-9	648028022

Table S4. List of Oxyphotobacteria, Melainabacteria and outgroups using universal single copy bacterial marker gene sets

Bacterial and archaeal genomes used to produce **Figs S3 and S4**. Organisms in black were used to produce phylogenetic trees using both the 38 marker and 83 marker sets. Organisms in blue were used for phylogenetic trees made with the 83 marker set only and organisms in red were used for phylogenetic trees made with the 38 marker set.

Phylum	Organism name	IMG/NCBI accession
Acidobacteria	*Acidobacterium capsulatum* ATCC 51196	643692001
Acidobacteria	*Granulicella mallensis* MP5ACTX8	648276601
Acidobacteria	*Korebacter versatilis* Ellin345	637000001
Acidobacteria	*Solibacter usitatus* Ellin6076	639633060
Acidobacteria	*Terriglobus saanensis* SP1PR4	649633100
Actinobacteria	*Acidimicrobium ferrooxidans* ICP, DSM 10331	644736322
Actinobacteria	*Atopobium parvulum* IPP 1246, DSM 20469	644736327
Actinobacteria	*Bifidobacterium longum* DJO10A	642555107
Actinobacteria	*Conexibacter woesei* ID131577, DSM 14684	646311917
Actinobacteria	*Corynebacterium efficiens* YS-314	644736345
Actinobacteria	*Cryptobacterium curtum* 12-3, DSM 15641	644736346
Actinobacteria	*Eggerthella lenta* VPI 0255, DSM 2243	644736358
Actinobacteria	*Gordonibacter pamelaeae* 7-10-1-bT, DSM 19378	650377943
Actinobacteria	*Leifsonia xyli xyli* CTCB07	637000149
Actinobacteria	*Micrococcus luteus* Fleming NCTC 2665	644736390
Actinobacteria	*Microlunatus phosphovorus* NM-1	650716058
Actinobacteria	*Micromonospora* sp. L5	649633069
Actinobacteria	*Propionibacterium freudenreichii shermanii* CIRM-BIA1	649633084
Actinobacteria	*Rhodococcus jostii* RHA1	637000234
Actinobacteria	*Rubrobacter xylanophilus* DSM 9941	637000248
Actinobacteria	*Streptomyces scabiei* 87.22	646564576
Actinobacteria	*Thermobifida fusca* YX	637000319
Aquificae	*Aquifex aeolicus* VF5	637000010
Aquificae	*Desulfurobacterium thermolithotrophum* BSA, DSM 11699	649633039
Aquificae	*Hydrogenivirga* sp. 128-5-R1-1	641380441
Aquificae	*Hydrogenobacter thermophilus* TK-6, DSM 6534	646311936
Aquificae	*Hydrogenobaculum* sp. SN	647000261
Aquificae	*Persephonella marina* EX-H1	643692030
Aquificae	*Sulfurihydrogenibium azorense* Az-Fu1	643692050
Aquificae	*Sulfurihydrogenibium* sp. YO3AOP1	642555165
Aquificae	*Thermocrinis albus* HI 11/12, DSM 14484	646564582
Aquificae	*Thermovibrio ammonificans* HB-1, DSM	649633104

	15698	
Bacteroidetes	*Bacteroides fragilis* 3_1_12	645058788
Bacteroidetes	*Bacteroidetes* sp. F0058	648861005
Bacteroidetes	*Capnocytophaga gingivalis* ATCC 33624	643886113
Bacteroidetes	*Chitinophaga pinensis* UQM 2034, DSM 2588	644736340
Bacteroidetes	*Chryseobacterium gleum* F93, ATCC 35910	643886082
Bacteroidetes	*Croceibacter atlanticus* HTCC2559	648028020
Bacteroidetes	*Cytophaga hutchinsonii* ATCC 33406	637000087
Bacteroidetes	*Flavobacterium johnsoniae* UW101, ATCC 17061	644736369
Bacteroidetes	*Kordia algicida* OT-1	641380434
Bacteroidetes	*Leadbetterella byssophila* 4M15, DSM 17132	649633063
Bacteroidetes	*Parabacteroides merdae* ATCC 43184	640963016
Bacteroidetes	*Porphyromonas asaccharolytica* PR426713P-I	649989985
Bacteroidetes	*Porphyromonas endodontalis* ATCC 35406	643886148
Bacteroidetes	*Porphyromonas gingivalis* ATCC 33277	642555148
Bacteroidetes	*Prevotella melaninogenica* ATCC 25845	648028051
Bacteroidetes	*Prevotella tannerae* ATCC 51259	645951840
Bacteroidetes	*Psychroflexus torquis* ATCC 700755	638341165
Bacteroidetes	*Sphingobacterium spiritivorum* ATCC 33300	643886135
Chlamydiae	*Chlamydia muridarum* MoPn / Nigg	637000062
Chlamydiae	*Chlamydia trachomatis* A/HAR-13	637000063
Chlamydiae	*Chlamydophila abortus* S26/3	637000065
Chlamydiae	*Chlamydophila caviae* GPIC	637000066
Chlamydiae	*Chlamydophila felis* Fe/C-56	637000067
Chlamydiae	*Chlamydophila pecorum* E58	650716022
Chlamydiae	*Chlamydophila pneumoniae* AR39	637000068
Chlamydiae	*Chlamydophila psittaci* 01DC11	651053012
Chlamydiae	*Parachlamydia acanthamoebae* Hall's coccus	647000287
Chlamydiae	*Simkania negevensis* Z	650716085
Chlamydiae	*Waddlia chondrophila* WSU 86-1044	646564588
Chlorobi	*Chlorobaculum parvum* NCIB 8327	642555120
Chlorobi	*Chlorobium chlorochromatii* CaD3	637000072
Chlorobi	*Chlorobium limicola* DSM 245	642555121
Chlorobi	*Chlorobium phaeobacteroides* BS1	642555122
Chlorobi	*Chlorobium phaeovibrioides* DSM 265	640427130
Chlorobi	*Chlorobium tepidum* TLS	637000073
Chlorobi	*Chloroherpeton thalassium* ATCC 35110	642555123
Chlorobi	*Pelodictyon luteolum* DSM 273	637000205
Chlorobi	*Pelodictyon phaeoclathratiforme* BU-1	642555146
Chlorobi	*Prosthecochloris aestuarii* SK413, DSM 271	642555149
Chloroflexi	*Anaerolinea thermophila* UNI-1	649633005
Chloroflexi	*Chloroflexus aggregans* DSM 9485	643348527
Chloroflexi	*Chloroflexus aurantiacus* J-10-fl	641228485
Chloroflexi	*Dehalococcoides ethenogenes* 195	637000089
Chloroflexi	*Dehalococcoides* sp. BAV1	640427111

Chloroflexi	*Dehalogenimonas lykanthroporepellens* BL-DC-9	648028022
Chloroflexi	*Herpetosiphon aurantiacus* DSM 785	641228494
Chloroflexi	*Oscillochloris trichoides* DG6	649989977
Chloroflexi	*Roseiflexus castenholzii* HLO8, DSM 13941	640753047
Chloroflexi	*Roseiflexus* sp. RS-1	640427139
Chloroflexi	*Sphaerobacter thermophilus* 4ac11, DSM 20745	646311953
Chloroflexi	*Thermobaculum terrenum* YNP1, ATCC BAA-798	646311962
Chloroflexi	*Thermomicrobium roseum* DSM 5159	643348582
Chrysiogenetes	*Desulfurispirillum indicum* S5, DSM 22839	649633038
Cyanobacteria	*Acaryochloris marina* MBIC11017	641228474
Cyanobacteria	ACD20	2541046958
Cyanobacteria	*Anabaena variabilis* ATCC 29413	646564504
Cyanobacteria	*Arthrospira maxima* CS-328	642979357
Cyanobacteria	*Arthrospira platensis* NIES-39	650377906
Cyanobacteria	*Arthrospira* sp. PCC 8005	648276619
Cyanobacteria	*Candidatus* Caenarcanophila bioreactus	2523533519
Cyanobacteria	*Candidatus* Gastroanaerophila phascolarctos	2523533519
Cyanobacteria	*Candidatus* Obscuribacter phosphatis	2541046960
Cyanobacteria	*Crocosphaera watsonii* WH 8501	638341074
Cyanobacteria	cyanobacterium UCYN-A	646311970
Cyanobacteria	*Cyanobium* sp. PCC 7001	647533126
Cyanobacteria	*Cyanothece* sp. BH68, ATCC 51142	641522622
Cyanobacteria	*Cyanothece* sp. PCC 7424	643348533
Cyanobacteria	*Cyanothece* sp. PCC 7425	643348534
Cyanobacteria	*Cyanothece* sp. PCC 7822	648028021
Cyanobacteria	*Cyanothece* sp. PCC 8801	643348535
Cyanobacteria	*Cylindrospermopsis raciborskii* CS-505	647000233
Cyanobacteria	*Gloeobacter violaceus* PCC 7421	637000121
Cyanobacteria	*Lyngbya* sp. CCY 8106	639857035
Cyanobacteria	MEL_A1	2541046959
Cyanobacteria	MEL_B1	2541046956
Cyanobacteria	MEL_B2	2541046940
Cyanobacteria	MEL_C1	2541046938
Cyanobacteria	MH_37	2522572068
Cyanobacteria	*Microcoleus chthonoplastes* PCC 7420	647533184
Cyanobacteria	*Microcystis aeruginosa* NIES-843	641522640
Cyanobacteria	*Nodularia spumigena* CCY9414	639857037
Cyanobacteria	*Nostoc azollae* 0708	648028001
Cyanobacteria	*Nostoc punctiforme* PCC 73102	642555144
Cyanobacteria	*Nostoc* sp. PCC 7120	637000199
Cyanobacteria	*Oscillatoria* sp. PCC 6506	648276706
Cyanobacteria	*Prochlorococcus marinus* AS9601	640069321
Cyanobacteria	*Prochlorococcus marinus marinus* CCMP1375	637000213
Cyanobacteria	*Prochlorococcus marinus* MIT 9211	641228501

Cyanobacteria	*Prochlorococcus marinus* MIT 9313	637000211
Cyanobacteria	*Prochlorococcus marinus* MIT 9515	640069324
Cyanobacteria	*Prochlorococcus marinus* NATL2A	637000212
Cyanobacteria	*Prochlorococcus marinus pastoris* CCMP1986	637000214
Cyanobacteria	*Prochlorococcus* sp. CC9311	637000309
Cyanobacteria	*Prochlorococcus* sp. CC9605	637000310
Cyanobacteria	*Prochlorococcus* sp. CC9902	637000311
Cyanobacteria	*Prochlorococcus* sp. WH 7803	640427149
Cyanobacteria	*Prochlorococcus* sp. WH8102	637000314
Cyanobacteria	*Raphidiopsis brookii* D9	647000303
Cyanobacteria	*Synechococcus elongatus* PCC 6301	637000307
Cyanobacteria	*Synechococcus* sp. CC9616	2514885022
Cyanobacteria	*Synechococcus* sp. CC9616	2517093019
Cyanobacteria	*Synechococcus* sp. JA-2-3B	637000312
Cyanobacteria	*Synechococcus* sp. JA-3-3Ab	637000313
Cyanobacteria	*Synechococcus* sp. PCC 7002	641522654
Cyanobacteria	*Synechococcus* sp. RCC307	640427148
Cyanobacteria	*Synechocystis* sp. PCC 6803	637000315
Cyanobacteria	*Thermosynechococcus elongatus* BP-1	637000320
Cyanobacteria	*Trichodesmium erythraeum* IMS101	637000329
Cyanobacteria	Zag_1	2523533517
Cyanobacteria	Zag_111	2531839741
Deferribacteres	*Calditerrivibrio nitroreducens* Yu37-1, DSM 19672	649633026
Deferribacteres	*Deferribacter desulfuricans* SSM1, DSM 14783	646564525
Deferribacteres	*Denitrovibrio acetiphilus* N2460, DSM 12809	646564527
Dictyoglomi	*Dictyoglomus thermophilum* H-6-12, ATCC 35947	643348542
Dictyoglomi	*Dictyoglomus turgidum* DSM 6724	643348543
Elusimicrobia	*Candidatus* Endomicrobium sp. Rs-D17	642555172
Elusimicrobia	*Elusimicrobium minutum* Pei191	642555127
Firmicutes	*Acetohalobium arabaticum* Z-7288, DSM 5501	648028002
Firmicutes	*Clostridium thermocellum* ATCC 27405	640069309
Firmicutes	*Coprothermobacter proteolyticus* DSM 5265	643348530
Firmicutes	*Halothermothrix orenii* H 168	643348557
Firmicutes	*Mesoplasma florum* L1	637000158
Firmicutes	*Moorella thermoacetica* ATCC 39073	637000167
Firmicutes	*Mycoplasma mobile* 163K	637000180
Firmicutes	*Pelotomaculum thermopropionicum* SI	640427128
Firmicutes	*Syntrophothermus lipocalidus* DSM 12680	646564577
Fusobacteria	*Fusobacterium gonidiaformans* ATCC 25563	645951804
Fusobacteria	*Fusobacterium mortiferum* ATCC 9817	646206254
Fusobacteria	*Fusobacterium nucleatum nucleatum* ATCC 23726	647000254
Fusobacteria	*Fusobacterium periodonticum* ATCC 33693	645951848

Fusobacteria	*Fusobacterium* sp. 11_3_2	651324032
Fusobacteria	*Fusobacterium ulcerans* ATCC 49185	645951859
Fusobacteria	*Fusobacterium varium* ATCC 27725	646206275
Fusobacteria	*Leptotrichia buccalis* C-1013-b, DSM 1135	644736384
Fusobacteria	*Leptotrichia goodfellowii* F0264	647000268
Fusobacteria	*Leptotrichia hofstadii* F0254	645951860
Fusobacteria	*Sebaldella termitidis* ATCC 33386	646311952
Fusobacteria	*Streptobacillus moniliformis* 9901, DSM 12112	646311956
Gemmatimonadetes	*Gemmatimonas aurantiaca* T-27T	643692024
Lentisphaerae	*Lentisphaera araneosa* HTCC2155	640963040
Nitrospirae	*Thermodesulfovibrio yellowstonii* DSM 11347	643348581
Planctomycetes	*Blastopirellula marina* SH 106T, DSM 3645	638341020
Planctomycetes	*Candidatus* Kuenenia stuttgartiensis	642555116
Planctomycetes	*Gemmata obscuriglobus* UQM 2246	641736268
Planctomycetes	*Isosphaera pallida* IS1B, ATCC 43644	649633058
Planctomycetes	*Pirellula staleyi* DSM 6068	646311948
Planctomycetes	*Planctomyces brasiliensis* IFAM 1448, DSM 5305	649633083
Planctomycetes	*Planctomyces limnophilus* Mu 290, DSM 3776	646564559
Planctomycetes	*Planctomyces maris* DSM 8797	640963032
Planctomycetes	*Rhodopirellula baltica* SH 1	637000236
Proteobacteria	*Alcanivorax borkumensis* SK2	637000004
Proteobacteria	*Anaeromyxobacter dehalogenans* 2CP-1	643348507
Proteobacteria	*Anaplasma phagocytophilum* HZ	637000009
Proteobacteria	*Arcobacter nitrofigilis* DSM 7299	646564506
Proteobacteria	*Azoarcus* sp. BH72	639633007
Proteobacteria	*Bartonella bacilliformis* KC583	639633009
Proteobacteria	*Bordetella bronchiseptica* RB50	637000032
Proteobacteria	*Brucella melitensis* ATCC 23457	643692012
Proteobacteria	*Burkholderia cenocepacia* AU 1054	637000046
Proteobacteria	*Caminibacter mediatlanticus* TB-2	640963039
Proteobacteria	*Campylobacter concisus* 13826	640753009
Proteobacteria	*Campylobacter fetus fetus* 82-40	639633016
Proteobacteria	*Campylobacter lari* RM2100	643692014
Proteobacteria	*Candidatus* Puniceispirillum marinum IMCC1322	646564516
Proteobacteria	*Cellvibrio japonicus* Ueda107	642555119
Proteobacteria	*Chromohalobacter salexigens* 1H11, DSM 3043	637000075
Proteobacteria	*Comamonas testosteroni* CNB-1	646564523
Proteobacteria	*Cupriavidus taiwanensis* LMG 19424	644736347
Proteobacteria	*Dechloromonas aromatica* RCB	637000088
Proteobacteria	*Desulfarculus baarsii* 2st14, DSM 2075	648028023
Proteobacteria	*Desulfobacterium autotrophicum* HRM2, DSM 3382	643692021

Proteobacteria	*Desulfohalobium retbaense* HR100, DSM 5692	644736349
Proteobacteria	*Desulfomicrobium baculatum* X, DSM 4028	644736350
Proteobacteria	*Desulfonatronospira thiodismutans* ASO3-1	643886196
Proteobacteria	*Desulfovibrio magneticus* RS-1	644736352
Proteobacteria	*Desulfovibrio vulgaris* Miyazaki F	643348539
Proteobacteria	*Desulfurivibrio alkaliphilus* AHT2	646564528
Proteobacteria	*Dichelobacter nodosus* VCS1703A	640427112
Proteobacteria	*Dinoroseobacter shibae* DFL-12, DSM 16493	641228491
Proteobacteria	*Erwinia amylovora* CFBP1430	646564531
Proteobacteria	*Erythrobacter litoralis* HTCC2594	637000103
Proteobacteria	*Escherichia coli* 55989	643348544
Proteobacteria	*Francisella philomiragia philomiragia* ATCC 25017	641522628
Proteobacteria	*Geobacter sulfurreducens* KN400	648231707
Proteobacteria	*Geobacter uraniireducens* Rf4	640427115
Proteobacteria	*Haliangium ochraceum* SMP-2, DSM 14365	646311933
Proteobacteria	*Helicobacter felis* CS1, ATCC 49179	649633054
Proteobacteria	*Helicobacter mustelae* ATCC 43772	646564537
Proteobacteria	*Helicobacter pullorum* MIT 98-5489	643886218
Proteobacteria	*Herbaspirillum seropedicae* SmR1	648028033
Proteobacteria	*Hyphomicrobium denitrificans* ATCC 51888	648028034
Proteobacteria	*Hyphomonas neptunium* ATCC 15444	637000135
Proteobacteria	*Kangiella koreensis* SW-125, DSM 16069	644736377
Proteobacteria	*Laribacter hongkongensis* HLHK9	643692026
Proteobacteria	*Lawsonia intracellularis* PHE/MN1-00	637000145
Proteobacteria	*Legionella longbeachae* NSW150	648028038
Proteobacteria	*Magnetococcus* sp. MC-1	639633036
Proteobacteria	*Magnetospirillum magneticum* AMB-1	637000155
Proteobacteria	*Mariprofundus ferrooxydans* PV-1	639857004
Proteobacteria	*Methylobacterium populi* BJ001	642555139
Proteobacteria	*Myxococcus fulvus* HW-1	650716065
Proteobacteria	*Nautilia profundicola* Am-H	643692029
Proteobacteria	*Neisseria lactamica* 020-06	649633075
Proteobacteria	*Neorickettsia risticii* Illinois	644736395
Proteobacteria	*Nitratifractor salsuginis* E9I37-1, DSM 16511	649633076
Proteobacteria	*Nitratiruptor* sp. SB155-2	640753037
Proteobacteria	*Nitrobacter winogradskyi* Nb-255	637000193
Proteobacteria	*Nitrosococcus watsoni* C-113	648028046
Proteobacteria	*Nitrosomonas europaea* ATCC 19718	637000195
Proteobacteria	*Nitrosospira multiformis* ATCC 25196	637000197
Proteobacteria	*Paracoccus denitrificans* PD1222	639633048
Proteobacteria	*Parvibaculum lavamentivorans* DS-1	640753040
Proteobacteria	*Parvularcula bermudensis* HTCC2503	648028050
Proteobacteria	*Pasteurella multocida multocida* Pm70	637000203
Proteobacteria	*Pelobacter carbinolicus* DSM 2380	637000204
Proteobacteria	*Pelobacter propionicus* DSM 2379	639633050

Proteobacteria	*Phenylobacterium zucineum* HLK1	642555147
Proteobacteria	*Polaromonas* sp. JS666	637000208
Proteobacteria	*Pseudomonas putida* BIRD-1	650377963
Proteobacteria	*Psychrobacter* sp. PRwf-1	640427134
Proteobacteria	*Pusillimonas* sp. T7-7	650716078
Proteobacteria	*Ralstonia pickettii* 12D	644736400
Proteobacteria	*Rhizobium rhizogenes* K84	643348504
Proteobacteria	*Rickettsia bellii* OSU 85-389	640753044
Proteobacteria	*Shewanella amazonensis* SB2B	639633057
Proteobacteria	*Starkeya novella* DSM 506	648028054
Proteobacteria	*Sulfurimonas autotrophica* OK10, DSM 16294	648028058
Proteobacteria	*Sulfurospirillum deleyianum* 5175, DSM 6946	646311960
Proteobacteria	*Syntrophobacter fumaroxidans* MPOB	639633063
Proteobacteria	*Syntrophus aciditrophicus* SB	637000317
Proteobacteria	*Thiobacillus denitrificans* ATCC 25259	637000324
Proteobacteria	*Thiomonas intermedia* K12	646564585
Proteobacteria	*Tolumonas auensis* TA 4, DSM 9187	643692052
Proteobacteria	*Vibrio furnissii* 2510/74, NCTC 11218	650377984
Proteobacteria	Wolbachia endosymbiont of Culex quinquefasciatus Pel	642555168
Proteobacteria	*Yersinia pseudotuberculosis* IP 31758	640753060
Spirochaetes	*Borrelia hermsii* DAH	642555108
Spirochaetes	*Borrelia spielmanii* A14S	642791612
Spirochaetes	*Borrelia valaisiana* VS116	641736181
Spirochaetes	*Brachyspira murdochii* 56-150, DSM 12563	646564514
Spirochaetes	*Leptospira borgpetersenii* sv Hardjo-bovis JB197	639633032
Spirochaetes	*Spirochaeta smaragdinae* SEBR 4228, DSM 11293	648028052
Spirochaetes	*Spirochaeta* sp. Buddy	650377973
Spirochaetes	*Treponema azotonutricium* ZAS-9	650716099
Spirochaetes	*Treponema brennaborense* DSM 12168	650716100
Spirochaetes	*Treponema phagedenis* F0421	649990026
Spirochaetes	*Treponema vincentii* ATCC 35580	645951869
Synergistetes	*Aminobacterium colombiense* ALA-1, DSM 12261	646564503
Synergistetes	*Jonquetella anthropi* E3_33 E1	645951855
Synergistetes	*Thermanaerovibrio acidaminovorans* Su883, DSM 6589	646311961
Thermi	*Deinococcus deserti* VCD115	643692020
Thermi	*Deinococcus geothermalis* DSM 11300	641228488
Thermi	*Deinococcus maricopensis* LB-34, DSM 21211	649633034
Thermi	*Deinococcus proteolyticus* MRP, DSM 20540	649633035
Thermi	*Deinococcus radiodurans* USUHS (R1)	637000092
Thermi	*Meiothermus ruber* 21, DSM 1279	646564545
Thermi	*Meiothermus silvanus* VI-R2, DSM 9946	646564546

Thermi	*Oceanithermus profundus* 506, DSM 14977	649633077
Thermi	*Thermus scotoductus* SA-01, ATCC 700910	649633105
Thermi	*Thermus thermophilus* HB27	637000322
Thermi	*Truepera radiovictrix* RQ-24, DSM 17093	646564586
Thermotogae	*Fervidobacterium nodosum* Rt17-B1	640753026
Thermotogae	*Kosmotoga olearia* TBF 19.5.1	644736379
Thermotogae	*Marinitoga piezophila* KA3	647533182
Thermotogae	*Mesotoga prima* MesG1.Ag.4.2	648276752
Thermotogae	*Petrotoga mobilis* SJ95	641228500
Thermotogae	*Thermosipho africanus* TCF52B	643348583
Thermotogae	*Thermosipho melanesiensis* BI429	640753057
Thermotogae	*Thermotoga lettingae* TMO	641228511
Thermotogae	*Thermotoga naphthophila* RKU-10	646311964
Thermotogae	*Thermotoga neapolitana* DSM 4359	643348584
Thermotogae	*Thermotoga petrophila* RKU-1	640427150
Verrucomicrobia	*Akkermansia muciniphila* ATCC BAA-835	642555104
Verrucomicrobia	*Chthoniobacter flavus* Ellin428	642791618
Verrucomicrobia	*Methylacidiphilum infernorum* V4	642555138
Verrucomicrobia	*Opitutus terrae* PB90-1	641522643
Verrucomicrobia	*Verrucomicrobiales* sp. DG1235	647533243
Verrucomicrobia	*Verrucomicrobium spinosum* DSM 4136	641736179
Firmicutes	*Anaerococcus prevotii* PC 1, DSM 20548	644736326
Firmicutes	*Lactobacillus gasseri* ATCC 33323	639633030
Firmicutes	*Listeria welshimeri* sv 6b, SLCC5334	639633035
Firmicutes	*Staphylococcus carnosus carnosus* TM300	643692037
Proteobacteria	*Acidithiobacillus caldus* SM-1	650716003
Proteobacteria	*Acidithiobacillus ferrooxidans* ATCC 23270	643348501
Proteobacteria	*Bacteriovorax marinus* SJ	650377909
Proteobacteria	*Bdellovibrio bacteriovorus* HD100	637000030
Proteobacteria	*Buchnera aphidicola* (Cinara tujafilina)	650716012
Proteobacteria	*Candidatus* Blochmannia floridanus	637000056
Proteobacteria	Wigglesworthia glossinidia endosymbiont of Glossina brevipalpis	637000338
Spirochaetes	*Candidatus* Cloacamonas acidaminovorans	642555115
Aquificae	*Sulfurihydrogenibium yellowstonense* SS-5	645058708
Caldiserica	*Caldisericum exile* AZM16c01	2513237181
Caldithrix	*Caldithrix abyssi* DSM 13497	2513237181
Chlorobi	*Chlorobium ferrooxidans* DSM 13031	638341060
Chrysiogenetes	*Chrysiogenes arsenatis* DSM 11915	2005520001
Crenarchaeota	*Acidilobus saccharovorans* 345-15	648028003
Crenarchaeota	*Desulfurococcus kamchatkensis* 1221n	643348540
Crenarchaeota	*Desulfurococcus mucosus* 07/1, DSM 2162	649633040
Crenarchaeota	*Hyperthermus butylicus* DSM 5456	640069314
Crenarchaeota	*Ignicoccus hospitalis* KIN4/I, DSM 18386	640753029
Crenarchaeota	*Ignisphaera aggregans* AQ1.S1, DSM 17230	648028035
Crenarchaeota	*Metallosphaera sedula* DSM 5348	640427120
Crenarchaeota	*Pyrobaculum calidifontis* JCM 11548	640069326
Crenarchaeota	*Staphylothermus marinus* F1, DSM 3639	640069332

Crenarchaeota	*Sulfolobus islandicus* HVE10/4	650377981
Crenarchaeota	*Sulfolobus tokodaii* 7, JCM 10545	638154519
Crenarchaeota	*Thermofilum pendens* Hrk 5	639633064
Crenarchaeota	*Thermoproteus uzoniensis* 768-20	650716098
Crenarchaeota	*Thermosphaera aggregans* M11TL, DSM 11486	646564583
Crenarchaeota	*Vulcanisaeta moutnovskia* 768-28	650377985
Cyanobacteria	*Arthrospira platensis* C1	2507262036
Cyanobacteria	*Cyanobacterium* sp JSC-1	2502171143
Cyanobacteria	*Fischerella* sp. JSC-11	2505679024
Cyanobacteria	*Oscillatoriales* sp. JSC-12	2510065010
Cyanobacteria	*Synechococcus elongatus* PCC 7942	2514885031
Cyanobacteria	*Synechococcus* sp. PE A4 65AY6A5	2512875021
Cyanobacteria	*Synechococcus* sp. WH 8016	2507262052
Euryarchaeota	*Aciduliprofundum boonei* T469	646564501
Euryarchaeota	*Archaeoglobus fulgidus* VC-16, DSM 4304	638154502
Euryarchaeota	*Archaeoglobus profundus* Av18, DSM 5631	646311906
Euryarchaeota	*Candidatus* Methanoregula boonei 6A8	640753014
Euryarchaeota	*Ferroglobus placidus* AEDII12DO, DSM 10642	646564534
Euryarchaeota	*Halalkalicoccus jeotgali* B3, DSM 18796	648028029
Euryarchaeota	*Haloarcula marismortui* ATCC 43049	638154503
Euryarchaeota	*Halobacterium* sp. NRC-1	638154504
Euryarchaeota	*Haloferax volcanii* DS2, ATCC 29605	646564536
Euryarchaeota	*Halogeometricum borinquense* PR3, DSM 11551	649633053
Euryarchaeota	*Halomicrobium mukohataei* arg-2, DSM 12286	644736372
Euryarchaeota	*Haloquadratum walsbyi* C23	651053028
Euryarchaeota	*Halorhabdus utahensis* AX-2, DSM 12940	644736373
Euryarchaeota	*Halorubrum lacusprofundi* ATCC 49239	643692025
Euryarchaeota	*Haloterrigena turkmenica* VKM B-1734, DSM 5511	646311934
Euryarchaeota	*Methanobacterium* sp. AL-21	650716052
Euryarchaeota	*Methanobrevibacter ruminantium* M1	646311943
Euryarchaeota	*Methanobrevibacter smithii* PS, ATCC 35061	640427121
Euryarchaeota	*Methanocaldococcus fervens* AG86	644736385
Euryarchaeota	*Methanocaldococcus infernus* ME	646564547
Euryarchaeota	*Methanocaldococcus jannaschii* DSM 2661	638154505
Euryarchaeota	*Methanocaldococcus* sp. FS406-22	646564548
Euryarchaeota	*Methanocaldococcus vulcanius* M7, DSM 12094	646311944
Euryarchaeota	*Methanocella paludicola* SANAE	646311945
Euryarchaeota	*Methanocella* sp. RC-I	640427153
Euryarchaeota	*Methanococcoides burtonii* DSM 6242	637000161
Euryarchaeota	*Methanococcus aeolicus* Nankai-3	640753034
Euryarchaeota	*Methanococcus maripaludis* C5	640069316
Euryarchaeota	*Methanococcus vannielii* SB	640753036

Euryarchaeota	*Methanococcus voltae* A3	646564549
Euryarchaeota	*Methanocorpusculum labreanum* Z	640069317
Euryarchaeota	*Methanoculleus marisnigri* JR1, DSM 1498	640069318
Euryarchaeota	*Methanohalobium evestigatum* Z-7303, DSM 3721	648028039
Euryarchaeota	*Methanohalophilus mahii* SLP, DSM 5219	646564550
Euryarchaeota	*Methanoplanus petrolearius* SEBR 4847, DSM 11571	648028040
Euryarchaeota	*Methanopyrus kandleri* AV19	638154507
Euryarchaeota	*Methanosaeta concilii* GP6	650716054
Euryarchaeota	*Methanosaeta thermophila* PT	639633038
Euryarchaeota	*Methanosarcina acetivorans* C2A	638154508
Euryarchaeota	*Methanosarcina barkeri* Fusaro, DSM 804	637000162
Euryarchaeota	*Methanosarcina mazei* Go1, DSM 3647	638154509
Euryarchaeota	*Methanosphaera stadtmanae* DSM 3091	637000163
Euryarchaeota	*Methanosphaerula palustris* E1-9c, DSM 19958	643348525
Euryarchaeota	*Methanospirillum hungatei* JF-1	637000164
Euryarchaeota	*Methanothermobacter marburgensis* Marburg DSM 2133	648028041
Euryarchaeota	*Methanothermobacter thermoautotrophicus* Delta H	638154510
Euryarchaeota	*Methanothermococcus okinawensis* IH1	650716055
Euryarchaeota	*Methanothermus fervidus* V24S, DSM 2088	649633067
Euryarchaeota	*Methanotorris igneus* Kol 5	650716056
Euryarchaeota	*Natrialba magadii* ATCC 43099	646564555
Euryarchaeota	*Natronomonas pharaonis* Gabara, DSM 2160	637000187
Euryarchaeota	*Picrophilus torridus* DSM 9790	638154512
Euryarchaeota	*Pyrococcus abyssi* GE5	638154514
Euryarchaeota	*Pyrococcus furiosus* DSM 3638	638154515
Euryarchaeota	*Pyrococcus horikoshii* OT3	638154516
Euryarchaeota	*Pyrococcus* sp. NA2	650716079
Euryarchaeota	*Pyrococcus yayanosii* CH1	650716080
Euryarchaeota	*Thermococcus barophilus* MP	650716096
Euryarchaeota	*Thermococcus gammatolerans* EJ3	644736411
Euryarchaeota	*Thermococcus kodakarensis* KOD1	638154520
Euryarchaeota	*Thermococcus onnurineus* NA1	643348580
Euryarchaeota	*Thermococcus sibiricus* MM 739	644736412
Euryarchaeota	*Thermococcus* sp. 4557	650716097
Euryarchaeota	*Thermoplasma acidophilum* DSM 1728	638154521
Euryarchaeota	*Thermoplasma volcanium* GSS1	638154522
Fibrobacteres	*Fibrobacter succinogenes succinogenes* S85	650377942
Firmicutes	*Eubacterium cylindroides* T2-87	650377935
Firmicutes	*Thermodesulfobium narugense* Na82, DSM 14796	2504756006
Fusobacteria	*Fusobacterium necrophorum funduliforme* 1_1_36S	2513237330
Fusobacteria	*Fusobacterium* sp. oral taxon 370 str. F0437	2513237336

Fusobacteria	*Ilyobacter polytropus* CuHBu1, DSM 2926	649633056
Fusobacteria	*Leptotrichia goodfellowii* LB 57, DSM 19756	2506520045
Fusobacteria	*Leptotrichia shahii* DSM 19757	2515154071
Fusobacteria	*Leptotrichia wadei* DSM 19758	2515154120
Korarchaeota	*Candidatus* Korarchaeum cryptofilum OPF8	641522611
Nanoarchaeota	*Nanoarchaeum equitans* Kin4-M	638154511
Nitrospirae	*Candidatus* Nitrospira defluvii	649633030
Nitrospirae	*Leptospirillum ferrooxidans* C2-3	2540341086
OP10	*Chthonomonas calidirosea* T49	2503242004
OP9	*Caldatribacterium* OP9-cSCG	APKF00000000
Saccharibacteria	*Candidatus* Saccharimonas aalborgensis	CP005957.1
Thaumarchaeota	*Cenarchaeum symbiosum* A	641522613
Thaumarchaeota	*Nitrosopumilus maritimus* SCM1	641228499
Verrucomicrobia	*Opitutaceae* sp. TAV2	640963002
WWE1	*Candidatus* Cloacamonas acidaminovorans	642555115

Table S5. Genes belong to pathways or assemblages from the Melainabacteria representatives from this study

Listed are the IMG gene IDs for each of the Melainabacteria that are deposited in IMG.

		Symbol	***Candidatus* Gastroanaerophila phascolarctos**	**Zag_1**	**Zag_111**	**MH_37**	***Candidatus* Obscuribacter phosphatis**	***Candidatus* Caenarcanophila bioreactus**
**EMP pathway**	**E.C number**							
Glucokinase	2.7.1.2	GK	2523622275	2523618351	2534657128<br>2534657723	2522811268	2541282499<br>2541283255	-
Glucose-6-phosphate isomerase	5.3.1.9	GPI	2523621784	2523618311	2534656686	2522811348	-	2554235349
Phosphofructokinase	2.7.1.11	PFK	-	-	2534656845	-	2541281805	2554235646
Fructose-bisphosphate aldolase	4.1.2.13	fbaA	2523623187	2523617884	2534656936	2522812428	2541285301	2554235409
Triosephosphate isomerase	5.3.1.1	TPI	2523623082	2523617328	2534658147	2522812616	2541284414	2554236361
Glyceraldehyde-3-phosphate dehydrogenase	1.2.1.12	GAP	2523622612	2523617047	2534657629	-	2541285214	2554236616
Phosphoglycerate kinase	2.7.2.3	PGK	2523621953	2523618679	2534656945	2522812014	2541285215	2554235911
Phosphoglycerate mutase	5.4.2.1	PGM	2523622862	2523618262	2534656858	2522813130	2541282187	2554235338
Enolase	4.2.1.11	ENO	2523623115	2523618038	2534656767	2522811435	2541282164	-
Pyruvate kinase	2.7.1.40	PK	2523622478	2523618904	2534656599	2522811367	2541285471	2554235818
**Fermentation**								
Acetaldehyde dehydrogenase/alcohol dehydrogenase	1.2.1.10/1.1.1.1	ALDH/ADH	2523622361	2523617074	2534658922	2522812406	2541282950	2554235808
Alcohol dehydrogenase, class IV	1.1.1.1	ADH	2523621842	2523617291	2534657773	2522812145	2541282501	2554235925
Lactate dehydrogenase	1.1.1.28	LDH	2523621888	2523618732	2534657873	2522811332	2541281883	2554236386
Pyruvate formate lyase	2.3.1.54	PFL	2523622898	-	2534656682	-	-	-

**Pentose phosphate pathway**								
Glucose-6-phosphate dehydrogenase	1.1.1.49	G6PD	-	-	-	-	2541282512	-
6-phosphogluconolactonase	3.1.1.31	PGLS	-	-	-	-	2541282514	-
6-phosphogluconate dehydrogenase	1.1.1.44	PGD	-	-	-	-	2541282515	-
Ribulose-5-phosphate 3-Epimerase	5.1.3.1	RPE	2523623171	2523619049	2534656104 2534657714	2522811657	2541282203	2554235427
Ribose-5-phosphate Isomerase	5.3.1.6	RPI	2523621712	2523619138	2534657230	2522811997	2541282259	2554235504
Transketolase	2.2.1.1	TKT	2523622052 2523621794	2523618017 2523618410	2534656618 2534657589	2522812113 2522812117 2522812264 2522812114 2522812118 2522813495	2541283291	2554236287 2554235334
Transaldolase	2.2.1.2	TAL	-	-	-	-	2541281904	2554236185 https://img.jgi.doe.gov/cgi-bin/er/main.cgi?section=GeneDetail&page=geneDetail&gene_oid=2534659562
**TCA cycle**								
Pyruvate dehydrogenase	1.2.4.1	PDK	-	-	-	-	2541283534 2541283535	-
Citrate synthase	2.3.3.1	CS	-	-	-	-	2541282238	-
Aconitase	4.2.1.3	ACO	-	-	-	-	2541284223	-

Isocitrate dehydrogenase	1.1.1.41	IDH	-	2523618070	2534657368	2522811358		2554236132
Isocitrate dehydrogenase	1.1.1.42	IDH	-	-	-	-	2541284187<br>2541284595	-
2-oxoacid:ferredoxin oxidoreductase	1.2.7.3	OFOR	2523622373<br>2523622374<br>2523622375<br>2523622658<br>2523622659<br>2523622660	2523617943<br>2523617944<br>2523617945	2534657734<br>2534657735<br>2534657736	2522812645<br>2522812646<br>2522812647	2541281896<br>2541281897<br>2541282395<br>2541282396	-
Succinyl-CoA synthetase	6.2.1.5	SCS	-	-	-	-	2541282222<br>2541282223	-
Succinate dehydrogenase	1.3.5.1	SDH	-	-	-	-	2541284229<br>2541284230	-
Fumarase	4.2.1.2	FH	-	-	-	-	2541282719	-
Malate dehydrogenase	1.1.1.37	MDH	-	-	-	-	2541284440	-
Phosphoenolpyruvate carboxykinase	4.1.1.32	PEPCK	-	-	-	-	2541283468	-
**Electron transport chain**								
Predicted nucleoside-diphosphate-sugar epimerases	1.6.99.3	-	-	-	-	-	2541282363<br>2541284393	-
NADH dehydrogenase subunit A	1.6.5.3	nuoA	-	-	-	-	2541285507	-
NADH dehydrogenase subunit B	1.6.5.3	nuoB	-	-	-	-	2541285508	-
NADH:ubiquinone oxidoreductase 27 kD subunit	1.6.5.3	nuoC	-	-	-	-	2541285509	-
NADH:ubiquinone oxidoreductase 49 kD subunit 7	1.6.5.3	nuoD	-	-	-	-	2541285510	-

NADH:ubiquinone oxidoreductase 24 kD subunit	1.6.5.3	nuoE	-	2523617688	2534659396	2522812754	-	2554235204	
NAD(P)-dependent iron-only hydrogenase diaphorase component flavavoprotein	1.6.5.3	nuoF	-	2523617687	2534659397	2522812755	-	2554235203	
NAD(P)-dependent iron-only hydrogenase catalytic subunit	1.6.5.3	nuoG	-	2523617686	2534659398	2522812756	-	2554235202	
NADH dehydrogenase subunit H	1.6.5.3	nuoH	-	-	-	-	2541285511	-	
NADH-quinone oxidoreductase, chain I	1.6.5.3	nuoI	-	-	-	-	2541285512	-	
NADH dehydrogenase subunit L	1.6.5.3	nuoL	-	-	-	-	2541281894	-	
Proton-translocating NADH-quinone oxidoreductase, chain M	-	nuoM	-	-	-	-	2541281893	-	
NADH dehydrogenase subunit N	1.6.5.3	nuoN	-	-	-	-	2541283929	-	
NADH:ubiquinone oxidoreductase subunit 5 (chain L)/Multisubunit Na+/H+ antiporter, MnhA subunit	1.6.5.3	ndhF	-	-	-	-	2541284457	-	
NADH:ubiquinone oxidoreductase subunit 6 (chain J)	1.6.5.3	ndhG	-	-	-	-	2541285513	-	
Succinate dehydrogenase subunit A	1.3.5.1/1.3.99.1	sdhA	-	-	-	-	2541284229	-	
Succinate dehydrogenase subunit B	1.3.5.1/1.3.99.1	sdhB	-	-	-	-	2541284230	-	

Cbb3-type cytochrome oxidase, cytochrome c subunit	-	ccoO	-	-	-	-	2541285061	-
Cbb3-type cytochrome oxidase, subunit 1	1.9.3.1	ccoN	-	-	-	-	2541285062	-
ATP synthase F1 subcomplex alpha subunit	3.6.3.1.4	atpA	2523622717	2523618879	2534659071	2522812078	2541284051	2554236353
ATP synthase F0 subcomplex A subunit	3.6.3.1.4	atpB	2523622712	2523619145	2534658915	2522812073	2541284046	2554235801
ATP synthase F1 subcomplex epsilon subunit	3.6.3.1.4	atpC	2523621799	2523618795	-	2522813083	2541283748	-
ATP synthase F1 subcomplex beta subunit	3.6.3.1.4	atpD	2523621800	2523618796	2534659931	2522813084	2541283746	2554236478
ATP synthase, F0 subunit C	3.6.3.1.4	atpE	2523622713	2523619144	2534659316	2522812074	2541284047	2554235285
F0-F1-type ATP synthase, subunit b	3.6.3.1.4	atpF	2523622714	2523619143	-	2522812075	2541284048<br>2541284049	-
ATP synthase F1 subcomplex gamma subunit	3.6.3.1.4	atpG	2523622718	2523618878	2534659807	2522812079	2541284052	2554235023
ATP synthase F1 subcomplex delta subunit	3.6.3.1.4	atpH	2523622716	-	-	2522812077	2541284050	-
ATP synthase F0 subcomplex C subunit	3.6.3.1.4	-	-	-	-	-	-	-
Inorganic pyrophosphatase	3.6.1.1	-	2523621727	2523617466	-	2522812545	2541285303	-
Polyphosphate kinase 1	2.7.4.1	ppk	-	-	-	-	2541282432	-
Vacuolar-type H(+) translocating pyrophosphatase	-	-	-	-	-	-	2541285109	-

**Flagella assembly**								
Flagella basal body P-ring formation protein FlgA		FlgA	-	-	-	-	-	-
flagellar basal-body rod protein FlgB		FlgB	2523621836	2523618479	2534656302	2522812505	-	-
flagellar basal-body rod protein FlgC		FlgC	2523621835	2523618478	2534656301	2522812504	-	-
Flagellar hook capping protein		FlgD	-	-	-	-	-	-
flagellar hook-basal body protein FlgE		FlgE	-	-	-	-	-	-
flagellar hook-basal body rod protein FlgF		FlgF	-	2523618481	2534656304	-	-	-
flagellar basal-body rod protein FlgG, Gram-negative bacteria		FlgG	2523621837<br>2523621838	2523618480	2534656303	2522812506	-	-
Flagellar basal body L-ring protein		FlgH	-	-	-	-	-	-
Flagellar basal-body P-ring protein		FlgI	-	-	-	-	-	-
Flagellar protein FlgJ		FlgJ	-	-	-	-	-	-
Flagellar hook-associated protein FlgK		FlgK	-	-	-	-	-	-
flagellar hook-associated protein 3		FlgL	-	-	-	-	-	-
FlgN		FlgN	-	-	-	-	-	-
Flagellar biosynthesis pathway, component FlhA		FlhA	2523622290	2523618233	2534658234	2522812376	-	2554236289
Flagellar biosynthesis pathway, component FlhB		FlhB	2523621565	2523617711	2534656770	2522811979	-	2554235321

Flagellin and related hook-associated proteins		FliC	-	-	-	-	-	-
Flagellar capping protein		FliD	-	-	-	-	-	-
flagellar hook-basal body complex protein FliE		FliE	2523622294	2523618229	-	2522812380	-	-
flagellar basal-body M-ring protein/flagellar hook-basal body protein (fliF)		FliF	-	-	-	-	-	-
Flagellar motor switch protein FliG		FliG	-	-	-	-	-	-
Flagellar biosynthesis/type III secretory pathway protein		FliH	-	-	-	-	-	-
type III secretion system ATPase, FliI/YscN (EC 3.6.3.15)		FliI	2523621472	2523618466 2523619149	2534657369	2522811726	-	2554236374
flagellar export protein FliJ		FliJ	2523621613	2523618709	-	2522811828	-	-
Flagellar hook-length control protein FliK		FliK	-	-	-	-	-	-
Flagellar basal body-associated protein		FliL	-	-	-	-	-	-
Flagellar motor switch protein FliM		FliM	-	-	-	-	-	-
Flagellar motor switch protein FliN		FliN/SpoA	-	-	-	-	-	-
Flagellar biosynthesis protein, FliO		FliO	-	-	-	-	-	-
Flagellar biosynthesis pathway, component FliP		FliP	2523622788	2523618755	2534657253	2522811903	-	2554235810
Flagellar biosynthesis pathway, component FliQ		FliQ	-	-	-	-	-	-

Flagellar biosynthesis pathway, component FliR		FliR	2523622786	2523618758	2534657251	2522812209	-	2554235468
flagellar biosynthetic protein FliS		FliS	-	-	-	-	-	-
Flagellar protiein FliT		FliT	-	-	-	-	-	-
Flagella motor component		MotA	-	-	-	-	-	-
Flagella motor protein		MotB	-	-	-	-	-	-
Flagellar motor protein		OmpA	-	-	2534656717	-	-	-
**Hydrogenases**								
[FeFe] hydrogenase H-cluster radical SAM maturase HydE	2.8.1.6	HydE	-	-	2534657619	2522813318	-	2554235784
iron-only hydrogenase maturation protein HydF	-	HydF	-	2523618604	2534656733	2522811229	-	2554235601
iron-only hydrogenase maturation protein HydG	-	HydG	-	2523617045	2534657108	2522813171	-	2554235630
[FeFe] hydrogenase, group B1/B3	-	-	-	-	2534657038	2522811239	-	-
Iron only hydrogenase large subunit, C-terminal domain	-	-	-	2523617266	2534657035	2522811236	-	-
NAD(P)-dependent iron-only hydrogenase diaphorase component flavoprotein	1.6.5.3	-	-	2523617687	2534657247	2522812755	-	2554235203
NAD(P)-dependent iron-only hydrogenase catalytic subunit	1.6.5.3	-	-	2523617686	2534657246	2522812756	-	2554235202
Ni,Fe-hydrogenase I small subunit	1.12.99.6	-	-	-	-	-	2541282828	-

Ni,Fe-hydrogenase I large subunit	1.12.99.6	-	-	-	-	-	2541282829	-
hydrogenase maturation protease	-	HycI	-	-	-	-	2541282830	-
Ni,Fe-hydrogenase III small subunit	-	-	2523623068	-	-	-	2541283385	-
Ni,Fe-hydrogenase III large subunit	-	-	2523623069	-	-	-	2541283386	-
Hydrogenase 4 membrane component (E)	1.-	-	2523623071	-	-	-	2541283388	-
Coenzyme F420-reducing hydrogenase, alpha subunit	-	-	-	-	-	-	2541281832	-
Coenzyme F420-reducing hydrogenase, beta subunit	-	-	-	-	2534657715	-	-	-
Coenzyme F420-reducing hydrogenase, delta subunit	-	-	-	-	-	-	2541285052	-
Coenzyme F420-reducing hydrogenase, gamma subunit	-	-	-	-	-	-	2541281831	-
Zn finger protein HypA/HybF (possibly regulating hydrogenase expression)	-	HypA/Hyb F	-	-	-	-	2541282821	-
Hydrogenase nickel incorporation protein HypB	-	HypB	-	-	-	-	2541282820	-
Hydrogenase maturation protein HypC	-	HypC	-	-	-	-	2541282822	-
Hydrogenase maturation protein HypD	-	HypD	-	-	-	-	2541282823<br>2541285008	-
Hydrogenase maturation	-	HypE	-	-	-	-	2541282825	-

protein, carbamoyl dehydratase HypE								
Hydrogenase maturation protein, carbamoyltransferase HypF	-	HypF	-	-	-	-	2541282826	-
**Polyphosphate metabolism**								
Polyphosphate kinase	-	-	-	-	-	2522813547	-	-
polyphosphate kinase 1	2.7.4.1	-	-	-	-	-	2541282432	-
polyphosphate kinase 2, PA0141 family	-	-	-	-	-	-	2541282023	-
Polyphosphate:AMP phosphotransferase	2.7.4.-	-	-	-	-	-	2541284980	-
Guanosine polyphosphate pyrophosphohydrolases/synthetases	3.1.7.2	-	2523622599	2523617618	2534656623	-	2541284760	-
Exopolyphosphatase	3.6.1.11	-	-	-	-	-	2541282351	-
Adenylate kinase	2.7.4.3	-	2523623169	2523616999	2534657048	2522813109	2541282076	2554235454

Table S6. Genomes used to produce the flagella gene tree

Listed are the IMG taxon ID, the organism name and the phylum that the organism belongs to.

IMG taxon ID	Organism	Phylum
643692001	*Acidobacterium capsulatum* ATCC 51196	Acidobacteria
649633100	*Terriglobus saanensis* SP1PR4, DSM 23119	Acidobacteria
642555107	*Bifidobacterium longum* DJO10A	Actinobacteria
2508501106	*Mycobacterium rhodesiae* NBB3	Actinobacteria
2517434006	*Brevibacterium casei* S18	Actinobacteria
646564582	*Thermocrinis albus* HI 11/12, DSM 14484	Aquificae
643692050	*Sulfurihydrogenibium azorense* Az-Fu1	Aquificae
649633104	*Thermovibrio ammonificans* HB-1, DSM 15698	Aquificae
2511231141	*Alicyclobacillus acidocaldarius acidocaldarius* Tc-4-1	Bacillus
649633013	*Bacteroides salanitronis* BL78, DSM 18170	Bacteroides
640753008	*Bacteroides vulgatus* ATCC 8482	Bacteroides
637000065	*Chlamydophila abortus* S26/3	Chlamydia
637000067	*Chlamydophila felis* Fe/C-56	Chlamydia
646564588	*Waddlia chondrophila* WSU 86-1044	Chlamydia
637000073	*Chlorobium tepidum* TLS	Chlorobi
637000072	*Chlorobium chlorochromatii* CaD3	Chlorobi
642555122	*Chlorobium phaeobacteroides* BS1	Chlorobi
2508501111	*Herpetosiphon aurantiacus* DSM 785	Chloroflexi
649989977	*Oscillochloris trichoides* DG6	Chloroflexi
649633005	*Anaerolinea thermophila* UN-1	Chloroflexi
649633038	*Desulfurispirillum indicum* S5, DSM 22839	Chrysiogenetes
637000076	*Clostridium acetobutylicum* ATCC 824	Clostridia
2503508009	*Mahella australiensis* 50-1 BON, DSM 15567	Clostridia
641522632	*Heliobacterium modesticaldum* Ice1	Clostridia
649633052	*Halanaerobium hydrogenoformans*	Clostridia
640427120	*Metallosphaera sedula* DSM 5348	Crenarchaeota
641228499	*Nitrosopumilus maritimus* SCM1	Crenarchaeota
648028062	*Vulcanisaeta distributa* DSM 14429	Crenarchaeota
2503982047	*Anabaena cylindrica* PCC 7122	Cyanobacteria
639857037	*Nodularia spumigena* CCY9414	Cyanobacteria
637000313	*Synechococcus* sp. JA-3-3Ab	Cyanobacteria
637000121	*Gloeobacter violaceus* PCC 7421	Cyanobacteria
2523533517	Zag_1	Cyanobacteria
2531839741	Zag_111	Cyanobacteria
2523533519	*Ca.* Gastranaerophilus phascolarctosicola	Cyanobacteria
2522572068	MH_37	Cyanobacteria
2541046959	Mel_A1	Cyanobacteria

2541046956	Mel_B1	Cyanobacteria
2541046940	Mel_B2	Cyanobacteria
2541046938	Mel_C1	Cyanobacteria
2531839742	*Ca.* Caenarcanum bioreactoricola	Cyanobacteria
2541046960	*Ca.* Obscuribacter phosphatis	Cyanobacteria
2541046958	ACD20	Cyanobacteria
643348543	*Dictyoglomus turgidum* DSM 6724	Dictyoglomi
643348542	*Dictyoglomus thermophilum* H-6-12, ATCC 35947	Dictyoglomi
642555127	*Elusimicrobium minutum* Pei191	Elusimicrobia
642555172	*Candidatus* Endomicrobium sp. Rs-D17	Elusimicrobia
644736373	*Halorhabdus utahensis* AX-2	Euryarcheota
648028040	*Methanoplanus petrolearius* SEBR 4847	Euryarcheota
638154522	*Thermoplasma volcanium* GSS1	Euryarcheota
650377942	*Fibrobacter succinogenes succinogenes* S85	Fibrobacter
637000117	*Fusobacterium nucleatum nucleatum* ATCC 25586	Fusobacteria
646311952	*Sebaldella termitidis* ATCC 33386	Fusobacteria
644736384	*Leptotrichia buccalis* C-1013-b, DSM 1135	Fusobacteria
643692024	*Gemmatimonas aurantiaca* T-27T	Gemmatimonadetes
640963040	*Lentisphaera araneosa* HTCC2155	Lentisphaerae
650633000	*Victivallis vadensis* ATCC BAA-548	Lentisphaerae
638154511	*Nanoarchaeum equitans* Kin4-M	Nanoarchaeota
2540341086	*Leptospirillum ferrooxidans* C2-3	Nitrospirae
637000236	*Rhodopirellula baltica* SH 1	Planctomycetes
649633083	*Planctomyces brasiliensis* IFAM 1448, DSM 5305	Planctomycetes
641736268	*Gemmata obscuriglobus* UQM 2246	Planctomycetes
643692004	*Azotobacter vinelandii* DJ, ATCC BAA-1303	Proteobacteria
649633004	*Alicycliphilus denitrificans* BC	Proteobacteria
650716078	*Pusillimonas* sp. T7-7	Proteobacteria
637000241	*Rhodospirillum rubrum* S1, ATCC 11170	Proteobacteria
646311920	*Dickeya dadantii* Ech586	Proteobacteria
644736355	*Dickeya zeae* Ech1591	Proteobacteria
637000207	*Photorhabdus luminescens laumondii* TTO1	Proteobacteria
650377903	*Acinetobacter calcoaceticus* PHEA-2	Proteobacteria
643348518	*Borrelia duttonii* Ly	Spirochaetes
2506783010	*Leptonema illini* 3055, DSM 21528	Spirochaetes
2511231215	*Treponema pallidum pertenue* Gauthier	Spirochaetes
2505119043	*Thermovirga lienii* Cas60314, DSM 17291	Synergistetes
646311961	*Thermanaerovibrio acidaminovorans* Su883, DSM 6589	Synergistetes
645951855	*Jonquetella anthropi* E3_33 E1	Synergistetes
2508501115	*Deinococcus pimensis* KR-235	Thermi
646564545	*Meiothermus ruber* 21, DSM 1279	Thermi

2515154172	*Thermus igniterrae* ATCC 700962	Thermi
2505119042	*Thermodesulfatator indicus* CIR29812, DSM 15286	Thermodesulfobacteria
640427150	*Thermotoga petrophila* RKU-1	Thermotoga
2510065086	*Mesotoga prima* MesG1Ag4.2	Thermotoga
2519899531	*Thermotoga maritima* MSB8, DSM 3109	Thermotoga
2517572100	*Opitutaceae* sp. TAV2	Verrucomicrobia
641522643	*Opitutus terrae* PB90-1	Verrucomicrobia
642791618	*Chthoniobacter flavus* Ellin428	Verrucomicrobia

Table S7. Table of Pfams used to differentiate cell wall types and flagella assembly, and GI numbers for photosynthesis genes and (bacterio)chlorophyll biosynthesis genes

The PFAM numbers that were used to differentiate cell wall were obtained from Albertsen et al., 2013. The flagella assembly genes are outlined in Pallen & Matzke, 2006 and the the (bacterio)chlorophyll biosynthesis genes and cut-offs were obtained from Sousa et al., 2012.

Pfam/GI	Pfam description
PF04413	Glycos_transf_N – (kdotransferase)
PF02614	LpxK – Tetraacyldisaccharide-1-P 4'-kinase
PF02684	LpxB – Lipid-A-disaccharide synthetase
PF03331	LpxC – UDP-3-O-acyl N-acetylglycosamine deacetylase
PF04613	LpxD – UDP-3-O-[3-hydroxymyristoyl] glucosamine N-acyltransferase, LpxD
PF02472	ExbD – Biopolymer transport protein ExbD/TolR
PF07244	Surf_Ag_VNR – Surface antigen variable number repeat
PF03739	YjgP_YjgQ – Predicted permease YjgP/YjgQ family
PF01103	Bac_surface_Ag – Surface antigen
PF00263	Secretin – Bacterial type II and III secretion system protein
PF02321	OEP – Outer membrane efflux protein
PF03968	OstA – OstA-like protein
PF00593	TonB_dep_Rec – TonB dependent receptor
PF04166	PdxA – Pyridoxal phosphate biosynthesis protein PdxA
PF06835	Lipopolysaccharide-assembly, LptC-related
PF03740	PdxJ – Pyridoxal phosphate biosynthesis protein PdxJ
PF03548	LolA – Outer membrane lipoprotein carrier protein LolA
PF04052	TolB_N – TolB amino-terminal domain
PF04453	OstA_C – Organic solvent tolerance protein
PF02645	DegV – Uncharacterised protein, DegV family COG1307
PF05103	DivIVA – DivIVA protein
PF02650	HTH_WhiA – Sporulation Regulator WhiA C terminal domain
PF10298	WhiA_N – Sporulation Regulator WhiA N terminal
PF04472	DUF552 – Protein of unknown function (DUF552)
PF04203	Sortase – Sortase family
PF03816	LytR_cpsA_psr – Cell envelope-related transcriptional attenuator domain
PF09269	DUF1967 – Domain of unknown function (DUF1967)
PF01424	R3H – R3H domain
PF01618	MotA_ExbB – MotA/TolQ/ExbB proton channel family
PF13677	MotB_plug – Membrane MotB of proton-channel complex MotA/MotB
PF03963	FlgD – Flagellar hook capping protein – N-terminal region
PF00460<br>PF06429	Flg_bb_rod – Flagella basal body rod protein<br>Flg_bbr_C – Flagellar basal body rod FlgEFG protein C-terminal
PF02107	FlgH - Flagellar L-ring protein
PF02119	FlgI - Flagellar P-ring protein
PF00669<br>PF00700	Flagellin_N – Bacterial flagellin N-terminal helical region<br>Flagellin_C – Bacterial flagellin C-terminal helical region

PF02465<br>PF07195	FliD_N - Flagellar hook-associated protein 2 N-terminus<br>FliD_C – Flagellar hook-associated protein 2 C-terminus
PF02049	FliE – Flagellar hook-basal body complex protein FliE
PF01514<br>PF08345	YscJ_FliF – Secretory protein of YscJ/FliF family<br>YscJ_FliF_C – Flagellar M-ring protein C-terminal
PF01706	FliG_C - FliG C-terminal domain
PF02108	FliH – Flagellar assembly protein FliH
PF02050	FliJ – Flagellar FliJ protein
PF02154	FliM - Flagellar motor switch protein FliM
PF01052	SpoA - Surface presentation of antigens (SPOA)
PF00813	FliP – FliP family
PF01313	Bac_export_3 – Bacterial export proteins, family 3
PF01311	Bac_export_1 – Bacterial export proteins, family 1
PF02561	FliS – Flagellar protein FliS
PF00771	FHIPEP - FHIPEP protein family
PF01312	Bac_export_2 – FlhB HrpN YscU SpaS Family
189347628<br>21674769<br>37522897<br>77463857	bchH
189346994<br>21674119<br>37520439<br>77463844	bchD
21674120<br>37521283<br>77463843	bchI
21674770<br>37523971<br>77463859	bchM
21674771<br>77463851	bchE
77463865	acsF
21673889	bciA
16331168	bciB
159462468	LPOR
189347668<br>21674961<br>37521938<br>77463855	bchN
21674960<br>37519784<br>77463856	bchB
189347666<br>21674959<br>37521939<br>77463858	bchL
